# Supplementary material for: Adult psychosocial outcomes of men and women who were looked-after or adopted as children: prospective observational study
Source: BMJ Open. 2018 Feb 8;8(2):e019095. doi: 10.1136/bmjopen-2017-019095 (PMC5829744; doi:10.1136/bmjopen-2017-019095)
Supplement: Supplementary file 1 [file bmjopen-2017-019095supp001.pdf]

Supplementary Tables

Table A: Social network questionnaire items in ALSPAC

Table B: Social support questionnaire items in ALSPAC

Table C: Summary of missing data

Table D: Summary of childhood and adolescent measures by care status for women and men

Tables E-I: Results for sensitivity analyses (complete case, pregnancy time-point outcomes only)

Social network and support measurement scales in ALSPAC

Below the items which comprise the social network scale and the social support scale are listed, along with the response options. Note that the ALSPAC data dictionary provides further details on these scales (see 'Mother Questionnaire D' document) and on all ALSPAC measures, and can be downl oaded from the ALSPAC website: http://www.bristol.ac.uk/alspac/researchers/

Table A: Summary of ALSPAC social network questionnaire items

Questionnaire Items	Response Options
1. How many of your relatives and your partner's relatives do you see at least twice a year?	None, 1, 2-4, >4
2. About how many friends do you have?	None, 1, 2-4, >4
3. Overall, would you say you belong to a close circle of friends?	Yes, No
4. How many people are there that you can talk to about personal problems?	None, 1, 2-4, >4
5. How many people talk to you about their personal problems or their private feelings?	None, 1, 2-4, >4
6. If you have to make an important decision, how many people are there with whom you can discuss it?	None, 1, 2-4, >4
7. How many people are there among your family and friends from whom you could borrow £100 if you needed to?	None, 1, 2-4, >4
8. How many of your family and friends would help you in times of trouble?	None, 1, 2-4, >4
9. During the last month, how many times did you get together with one or more friends?	None, 1, 2-4, >4
10. During the last month, how many times did you get together with one or more of your relatives or your partner's relatives?	None, 1, 2-4, >4

Table B: Summary of ALSPAC social support questionnaire items

Questionnaire Items	Response Options
1. I have no one to share my feelings with	Exactly feel, often feel, sometimes feel, never feel
2. My partner provides the emotional support I need	Exactly feel, often feel, sometimes feel, never feel
3. There are other pregnant women with whom I can share my experiences	Exactly feel, often feel, sometimes feel, never feel
4. I believe in moments of difficulty my neighbours would help me	Exactly feel, often feel, sometimes feel, never feel
5. I'm worried that my partner might leave me	Exactly feel, often feel, sometimes feel, never feel
6. There is always someone with whom I can share my happiness and excitement about my pregnancy	Exactly feel, often feel, sometimes feel, never feel
7. If I feel tired I can rely on my partner to take over	Exactly feel, often feel, sometimes feel, never feel
8. If I was in financial difficulty I know my family would help if they could	Exactly feel, often feel, sometimes feel, never feel
9. If I was in financial difficulty I know my friends would help of they could	Exactly feel, often feel, sometimes feel, never feel
10. If all else fails I know the state will support and assist me	Exactly feel, often feel, sometimes feel, never feel

Table C: Summary of missing data

		N (%) missing	
		Women (100%=8775)	Men (100%=3654)
**Exposure variables**	Looked after status	588 (6.7)	0
	Adoption status	313 (3.6)	13 (0.4)
**Outcomes**			
(a) Pre-pregnancy (or pre-partner's pregnancy)	**Substance use**		
	Alcohol - frequency	182 (2.1)	99 (2.7)
	Smoking	145 (1.7)	142 (3.9)
	Cannabis	504 (5.7)	1771 (48.5)
	Ever had addiction	243 (2.8)	6 (0.2)
(b) Pregnancy (or partner's pregnancy)	**Mental health**		
	Depressive symptoms (EPDS)	799 (9.1)	134 (3.7)
	Anxiety symptoms (Crown-crisp)	884 (10.1)	134 (3.7)
	Ever had mental health problem	243 (2.8)	15 (0.4)
	**Social environment**		
	Social support	795 (9.1)	245 (6.7)
	Social network	422 (4.8)	188 (5.1)
(c) Child aged 5 years	**Substance use**		
	Alcohol - frequency	39 (0.4)	14 (0.4)
	Alcohol - binge	127 (1.5)	N/A
	Smoking	114 (1.3)	93 (2.6)
	Cannabis	52 (0.6)	47 (1.3)
	**Mental health**		
	Depressive symptoms (EPDS)	104 (1.2)	N/A
	Anxiety symptoms (Crown-crisp)	155 (1.8)	N/A
	Anxiety/nerves in past year	184 (2.1)	79 (2.2)
	Depression in past year	171 (1.9)	89 (2.4)
(d) Pregnancy (or partner's pregnancy) to child aged 5 years	Any criminal conviction	1681 (19.2)	1265 (34.6)
**Other**	Age	0	192 (5.3)
	Educational attainment	251 (2.9)	105 (2.9)
	Housing tenure	216 (2.5)	20 (0.6)
	Financial difficulties	481 (5.5)	115 (3.2)
	Social class	721 (8.2)	139 (3.8)
	Partner status	160 (1.8)	2 (0.1)
	Pregnancy with study child intentional	184 (2.1)	21 (0.6)
	Pregnancy intentions at 5 yrs	92 (1.1)	N/A
	Childhood happiness	93 (1.1)	44 (1.2)
	Pregnancy (own or partner's) pre 17yrs	272 (3.1)	88 (2.4)
	Suspended from school pre 17yrs	272 (3.1)	88 (2.4)
	In trouble with police pre 17yrs	272 (3.1)	88 (2.4)
	Number of schools attended	655 (7.5)	900 (24.6)

Table D: Summary of childhood and adolescent measures by care status for women and men

		Women			Men		
		Reference	Looked After	Adopted	Reference	Looked After	Adopted
Highest childhood happiness	Very happy (%)	75.4 (74.4-76.3)	46.9 (38.4-55.3)	72.9 (67.0-78.8)	77.9 (76.5-79.3)	61.9 (48.0-75.8)	77.2 (68.3-86.2)
Number of schools attended by 16 yrs	0-2 (%)	43.3 (42.2-44.4)	16.9 (10.0-23.8)	39.8 (33.0-46.6)	42.0 (40.2-43.8)	20.4 (6.2-34.6)	40.0 (28.1-52.0)
	5+ (%)	7.9 (7.3-8.4)	29.7 (21.6-37.9)	12.5 (8.1-16.9)	8.1 (7.1-9.1)	22.3 (8.2-36.5)	9.3 (2.2-16.4)
Suspended from school pre 17 years	Yes (%)	2.4 (2.1-2.7)	16.1 (9.7-22.4)	6.0 (2.6-9.3)	6.2 (5.4-7.0)	15.2 (4.5-25.9)	12.3 (5.2-19.4)
In trouble with police pre 17 years	Yes (%)	3.2 (2.8-3.6)	14.4 (8.3-20.5)	8.3 (4.4-12.1)	13.5 (12.3-14.6)	41.3 (27.0-55.6)	30.1 (20.3-40.0)
Pregnancy pre 17 years	Yes (%)	5.6 (5.1-6.1)	22.4 (15.2-29.6)	8.3 (4.5-12.2)	1.7 (1.3-2.2)	n<5	n<5

Table E: Characteristics of the women by care status in childhood, n=7088 (complete case, cross-sectional sample)

		Not looked after or adopted N=6794	Looked After (not adopted) N=110	Adopted N=184
Maternal age at delivery	Mean (years)	28.6	27.2	27.7
	<=23 years (%)	12.5 (11.7-13.3)	25.5 (18.1-34.6)	15.8 (11.1-21.8)
	>=34 years (%)	14.1 (13.3-14.9)	10.0 (5.6-17.3)	9.8 (6.2-15.1)
Relationship status	Husband (%)	82.1 (81.2-83.0)	62.7 (53.2-71.4)	78.8 (72.2-84.1)
	Resident partner (%)	13.8 (13.0-14.6)	21.8 (15.0-30.6)	15.2 (10.7-21.2)
Parity	0 (%)	44.6 (43.4-45.8)	29.1 (21.3-38.4)	48.9 (41.7-56.2)
	3+ (%)	4.6 (4.1-5.1)	21.0 (14.2-29.7)	7.1 (4.1-11.8)
This pregnancy intentional	Yes (%)	75.1 (74.1-76.2)	53.6 (44.2-62.9)	71.2 (64.2-77.3)
Highest maternal education	Degree (%)	14.0 (13.2-14.9)	n<5	8.2 (5.0-13.2)
	Vocational/none (%)	23.8 (22.8-24.9)	53.6 (44.2-62.9)	22.8 (17.3-29.5)
Financial difficulties	Q1 (none) (%)	39.0 (37.9-40.2)	17.3 (11.2-25.7)	26.6 (20.7-33.5)
	Q4 (high) (%)	17.4 (16.5-18.3)	36.4 (27.8-45.9)	19.6 (14.4-26.0)
Housing tenure	Owned/mortgaged (%)	81.5 (80.6-82.4)	43.6 (34.6-53.2)	75.5 (68.8-81.3)
Lowest social class of self and partner	I&II	26.5 (25.4-27.5)	17.3 (11.2-25.7)	25.5 (19.7-32.4)
	IV&V	18.9 (18.0-19.8)	26.4 (18.9-35.5)	23.9 (18.3-30.7)

Table F: Characteristics of the men by care status in childhood, n=2820 (complete case, cross-sectional sample)

		Not looked after or adopted N=2662	Looked After (not adopted) N=68	Adopted N=90
Paternal age at 18wks gestation	Mean (years)	30.9	29.6	29.2
	<=23 years (%)	7.6 (6.7-8.7)	16.2 (9.1-27.2)	12.2 (30.3-50.6)
	>=34 years (%)	29.0 (27.3-30.7)	20.6 (12.4-32.2)	20.0 (12.9-29.7)
Relationship status	Husband (%)	81.1 (79.6-82.5)	72.1 (60.0-81.6)	73.3 (63.1-81.6)
Highest education	Degree (%)	18.2 (16.8-19.7)	n<5	14.4 (8.5-23.5)
	Vocational/none (%)	28.1 (26.4-29.8)	54.4 (42.2-66.1)	31.1 (22.3-41.6)
Financial difficulties	Q1 (none) (%)	37.0 (35.2-38.9)	14.7 (8.0-25.5)	26.7 (18.4-36.9)
	Q4 (high) (%)	18.8 (17.3-20.3)	44.1 (32.6-56.3)	24.4 (16.5-34.6)
Housing tenure	Owned/mortgaged (%)	78.4 (76.8-80.0)	47.1 (35.3-59.2)	67.8 (57.3-76.8)
Lowest social class of self and partner	I&II	26.3 (24.6-28.0)	17.6 (10.2-28.9)	26.7 (18.4-36.9)
	IV&V	21.3 (19.7-22.9)	32.4 (22.1-44.6)	22.2 (14.7-32.2)

Table G: Substance use pre-pregnancy by childhood care status for men and women (complete case, cross-sectional sample)

		Men			Women		
		%	OR (95% CI)		%	OR (95% CI)	
**Outcome**	**Care status**		Unadjusted	Adjusted[1]		Unadjusted	Adjusted[2]
Smoked regularly	None	57.0	Ref	Ref	29.2	Ref	Ref
	Looked After	76.5	2.5 (1.4-4.3)*	1.4 (0.8-2.6)	61.8	3.9 (2.7-5.8)*	1.9 (1.3-3.0)*
	Adopted	64.4	1.4 (0.9-2.1)	1.2 (0.7-1.8)	41.8	1.7 (1.3-2.3)*	1.6 (1.1-2.2)*
Drank alcohol every day	None	26.7	Ref	Ref	11.1	Ref	Ref
	Looked After	17.6	0.6 (0.3-1.1)	0.8 (0.4-1.5)	7.3	0.6 (0.3-1.3)	0.8 (0.4-1.6)
	Adopted	20.0	0.7 (0.4-1.2)	0.7 (0.4-1.2)	21.2	2.2 (1.5-3.1)*	2.4 (1.7-3.5)*
Used cannabis	None	10.1	Ref	Ref	3.5	Ref	Ref
	Looked After	11.8	1.2 (0.6-2.5)	0.8 (0.3-1.7)	9.1	2.7 (1.4-5.3)*	1.6 (0.8-3.3)
	Adopted	17.8	1.9 (1.1-3.4)*	1.5 (0.8-2.8)	8.2	2.4 (1.4-4.2)*	2.0 (1.1-3.7)*

[1]Adjusted for age, relationship status, education, financial difficulties, social class, housing tenure.

[2]Adjusted for age, relationship status, education, financial difficulties, social class, housing tenure, parity, pregnancy intentional.

*p<0.05

Table H: Mental health outcomes during the pregnancy by childhood care status for men and women (complete case, cross-sectional sample)

		Men			Women		
		%	**OR (95% CI)**		%	**OR (95% CI)**	
			Unadjusted	Adjusted[1]		Unadjusted	Adjusted[2]
High anxiety score (>6m, >9w)	None	10.6	Ref	Ref	10.4	Ref	Ref
	Looked After	14.7	1.5 (0.7-2.9)	1.3 (0.6-2.6)	26.4	3.1 (2.0-4.8)*	1.7 (1.1-2.7)*
	Adopted	11.1	1.1 (0.5-2.1)	1.0 (0.5-2.1)	19.0	2.0 (1.4-3.0)*	1.8 (1.2-2.6)*
High depression score (>8m, >13w)	None	12.7	Ref	Ref	8.6	Ref	Ref
	Looked After	22.1	2.0 (1.1-3.5)*	1.4 (0.8-2.6)	20.9	2.8 (1.8-4.5)*	1.5 (0.9-2.4)
	Adopted	12.2	1.0 (0.5-1.8)	0.9 (0.5-1.7)	16.3	2.1 (1.4-3.1)*	1.8 (1.2-2.8)*
Ever had mental health problem	None	8.3	Ref	Ref	10.0	Ref	Ref
	Looked After	17.6	2.4 (1.3-4.5)*	1.6 (0.8-3.2)	22.7	2.7 (1.7-4.2)*	1.7 (1.0-2.7)*
	Adopted	17.8	2.4 (1.4-4.2)*	2.2 (1.2-3.9)*	17.4	1.9 (1.3-2.8)*	1.8 (1.2-2.7)*

[1]Adjusted for age, relationship status, education, financial difficulties, social class, housing tenure.

[2]Adjusted for age, relationship status, education, financial difficulties, social class, housing tenure, parity, pregnancy intentional.

*p<0.05

Table I: Social support outcomes by childhood care status for men and women (complete case, cross-sectional sample)

		Men			Women		
	**Care status**	**%**	**OR (95% CI)**		**%**	**OR (95% CI)**	
			Unadjusted	Adjusted[1]		Unadjusted	Adjusted[2]
Low social support	None	13.9	Ref	Ref	8.0	Ref	Ref
	Looked After	26.5	2.2 (1.3-3.9)*	1.7 (1.0-3.0)	21.8	3.2 (2.0-5.1)*	1.5 (0.9-2.4)
	Adopted	15.6	1.1 (0.6-2.0)	1.1 (0.6-1.9)	9.8	1.2 (0.8-2.0)	1.1 (0.7-1.8)
Poor social network	None	15.8	Ref	Ref	8.6	Ref	Ref
	Looked After	22.1	1.5 (0.8-2.7)	1.0 (0.5-1.8)	24.5	3.5 (2.2-5.4)*	1.6 (1.0-2.6)*
	Adopted	15.6	1.0 (0.6-1.8)	0.9 (0.5-1.7)	13.6	1.7 (1.1-2.6)*	1.5 (1.0-2.4)

[1]Adjusted for age, relationship status, education, financial difficulties, social class, housing tenure.

[2]Adjusted for age, relationship status, education, financial difficulties, social class, housing tenure, parity, pregnancy intentional.

*$p<0.05$
