# Supplementary material for: Adult psychosocial outcomes of men and women who were looked-after or adopted as children: prospective observational study
Source: BMJ Open. 2018 Feb 8;8(2):e019095. doi: 10.1136/bmjopen-2017-019095 (PMC5829744; doi:10.1136/bmjopen-2017-019095)
Supplement: Author's manuscript [file bmjopen-2017-019095.draft_revisions.pdf]

BMJ Open

BMJ Open is committed to open peer review. As part of this commitment we make the peer review history of every article we publish publicly available.

When an article is published we post the peer reviewers’ comments and the authors’ responses online. We also post the versions of the paper that were used during peer review. These are the versions that the peer review comments apply to.

The versions of the paper that follow are the versions that were submitted during the peer review process. They are not the versions of record or the final published versions. They should not be cited or distributed as the published version of this manuscript.

BMJ Open is an open access journal and the full, final, typeset and author-corrected version of record of the manuscript is available on our site with no access controls, subscription charges or pay-per-view fees (http://bmjopen.bmj.com).

If you have any questions on BMJ Open’s open peer review process please email editorial.bmjopen@bmj.com

BMJ Open

Adult psychosocial outcomes of men and women who were looked-after or adopted as children: prospective observational study

Journal:	*BMJ Open*
Manuscript ID	bmjopen-2017-019095
Article Type:	Research
Date Submitted by the Author:	11-Aug-2017
Complete List of Authors:	Teyhan, Alison; Univ Bristol, Population Health Sciences, Bristol Medical School<br>Wijedasa, Dinithi; Univ Bristol, School for Policy Studies<br>Macleod, John; University of Bristol, Population Health Sciences, Bristol Medical School
**Primary Subject Heading**:	Epidemiology
Secondary Subject Heading:	Mental health, Smoking and tobacco, Addiction
Keywords:	Adoption, Looked-after child, Adult outcomes, Substance use, MENTAL HEALTH, ALSPAC

SCHOLARONE™
Manuscripts

Adult psychosocial outcomes of men and women who were looked-after or adopted as children: prospective observational study

A Teyhan[1*], D Wijedasa[2], J Macleod[1]

[1]Population Health Sciences, Bristol Medical School, University of Bristol, UK

[2]School of Policy Studies, University of Bristol, UK

*Corresponding author: alison.teyhan@bristol.ac.uk

For peer review only

Abstract

Objective

To investigate whether men and women who were looked-after (in public care) or adopted as children are at increased risk of adverse psychological and social outcomes.

Design, setting

Prospective observational study using the Avon Longitudinal Study of Parents and Children, which recruited pregnant women and their male partners in and around the city of Bristol, UK in the early 1990s.

Participants

8775 women and 3654 men who completed questionnaires at recruitment (mean age: women 29; men 32) and five years later.

Exposure

Childhood status: looked-after; adopted; not looked-after or adopted (reference group).

Outcomes

Substance use (alcohol, cannabis, tobacco) pre-pregnancy and five years later; if ever had addiction; anxiety and depression during pregnancy and five years later; if ever had mental health problem; social support during pregnancy; criminal conviction.

Results

For women, 2.7% were adopted and 1.8% had been looked-after; for men, 2.4% and 1.8% respectively. The looked-after group reported the poorest outcomes overall, but this was not a universal pattern, and there were gender differences. Smoking rates were high for both the looked-after (men 47%, women 58%) and adopted (men 44%, women 40%) groups relative to the reference group (both 28%). The looked-after group were at increased risk of a high depression score (men: 26% versus 11%, OR 2.9 [95% CI 1.5-5.6]; women: 24% versus 9%, 3.4 [2.2-5.0]). A high anxiety score was reported by 10% of the reference women, compared to 26% of those looked-after (3.0 [2.0-4.5]) and 17% of those adopted (1.8 [1.2-2.6]). Looked-after men and women reported the lowest social support, and criminal convictions and addiction were highest for looked-after men. Adjustment for adult socio-economic position generally attenuated associations for the looked-after group.

Conclusions

The needs of those who experience public care as children persist into adulthood. Health and social care providers should recognise this.

Strengths and Limitations

- The longitudinal, population-based cohort allows comparison of several psychosocial outcomes in adults who experienced being looked-after or adopted as children.
- Several of the outcomes are measured at two time-points five years apart, allowing the persistence or amelioration of disadvantage to be considered.
- We have several measures of adult socio-economic position, but do not have data on early life factors.
- The cohort only includes parents, which limits generalisability to the wider population of looked-after and adopted adults.
- Adoption and social care practices in England have changed since our participants were children, mainly in the 1960s and 1970s. Therefore the results may not be generalisable to younger adults.

For peer review only

1. Introduction

In the UK, the state has a duty to safeguard and promote the welfare of children, to protect them from maltreatment, and to promote their health and development (1). Surveillance by frontline services aims to identify children in need. Their families can be provided with additional support, but if this is inadequate to mitigate risk or to enable the child's needs to be met, parental responsibility may be taken on by the state. Reasons for this include abuse, neglect, family dysfunction, acute family stress, and disability. Almost 70,500 children in England are currently in the care of their local authorities (2), and one in 30 children experience out-of-home care by the age of 18 years (3).

The majority of 'looked-after' children in England today are placed in foster care (63% with 'stranger' carers, 11% with kin carers) (2). Children may be looked-after on a short or long-term basis, with some experiencing multiple periods of care (4). Within a period of care, a child may have several placements; recent statistics show that nearly a third experience one or more placement changes every year (2). A placement move can entail simultaneous changes to all developmental contexts, including family, school, neighbourhood and a change of social worker. Children who have already had a disadvantaged start in life can therefore have this further compounded by upheaval and instability once in care, resulting in substantial emotional, behavioural, social and educational problems (5-8).

Some children will remain in care until adulthood. For younger children in this situation, adoption is often the preferred long-term care model as it provides a greater level of permanence and a 'family for life' (2, 9). Children who grow up with adopted parents generally report higher levels of emotional security, sense of belonging, and well-being than those in long-term foster care (10). For those who age-out of the care system, the transition to adulthood can be fraught with difficulties. In England this happens at age 18, with the local authority continuing to provide support and advice until age 21, or 25 if in education or training (11). Beyond this, these young people often have little financial or social support. Their transition to adulthood is accelerated compared to their peers; they have to live independently, find employment, and manage their finances at a younger age, and without the support of a stable background (12).

Routine statistics and epidemiological studies show that children in care, both in the UK and elsewhere, do worse than their peers across many domains. Notably, they have lower educational attainment (13-18), poorer mental health (19-25), and are over-represented in the criminal justice system (26). They are at increased risk of many inter-linked adverse circumstances as they enter adulthood, including unemployment, homelessness, social isolation, drug use, self-harm, and imprisonment (8, 12, 27). These risks may be reduced if children are successfully adopted, however some studies have found that adopted children have more emotional, behavioural, and academic problems than their non-adopted peers (28), and experience more bullying in adolescence (29) .

Relatively few studies have considered outcomes beyond young adulthood, or how any adversities persist or change over time. In this study, we use data from a longitudinal, population-based cohort to examine outcomes at two time-points in adults who were

For peer review only

looked-after or adopted as children. We also consider the role of gender and adult socio-economic position. We chose outcomes which relate to four key areas in which looked-after children or young care leavers are known to experience increased risk: mental health difficulties, substance use, poor social support, and criminal conviction.

2. Methods

Sample

The Avon Longitudinal Study of Parents and Children (ALSPAC) recruited 14,541 pregnant women living in a defined area in and around the city of Bristol, UK in 1991-1992. The women have been sent regular postal questionnaires ever since. The women's partners have also completed questionnaires; these were distributed via the women. More details on ALSPAC are available in the cohort profiles (30, 31) and the searchable data dictionary (www.bristol.ac.uk/alspac/researchers/). The main results presented in this study are based on 8775 women and 3654 men who returned questionnaires during the pregnancy and when the study child was aged 5 years. We only include men who consistently reported being the father of the child to ensure we have the same man at both time-points.

Exposure measures

Experiences of being in care or adopted were reported via questionnaires administered during the pregnancy: adopted when aged <18 years (no, yes); ever been in the care of a local authority or voluntary agency (no; yes; unsure); ever stayed in a children's or residential home (no; <1 week; 1week – 1 month; 1-6 months; >6 months); ever stayed in a foster parents' home (no; yes). Those who responded yes to having been in care, or to having lived in a foster or children's home, were deemed to have been in care. A 3-category exposure variable was derived defining childhood care status: not looked-after or adopted; looked-after (not adopted); adopted. For the main analyses, the few individuals who reported that they were unsure if they had ever been looked-after (and who did not report that they had lived in children's home or foster care), were included in the looked-after group. A sensitivity analysis was also conducted excluding these individuals.

Main outcome measures

Substance use

Pre-pregnancy substance use was reported via questionnaires administered during the pregnancy with the study child: smoked regularly (no, yes); drank at least one unit of alcohol per day (no, yes) [1 UK unit is 8 grams of alcohol e.g. half a pint of lager (32)]; used cannabis in the 6 months before pregnancy (no, yes). Participants also reported if they had ever suffered from drug addiction or alcoholism (no, yes). Five years later, participants reported if they: currently smoked; had used cannabis in the past year; currently drank at least one unit of alcohol per day.

Mental health

For peer review only

During pregnancy, the participants reported if they had ever had schizophrenia, anorexia, severe depression, or other psychiatric problem: a variable was derived stating whether the respondent had ever had any of these psychiatric problems (no, yes). Symptoms of depression and anxiety were measured at 18 weeks gestation. Depressive symptoms were measured using the Edinburgh Postnatal Depression Scale. Although this measure was originally designed for use with postnatal women, the 10 items are not specific to women or this period and it has been validated for use at other times (33). Anxiety symptoms were measured by the 8 items of the anxiety subscale of the Crown-Crisp Experiential Index (34). Binary variables were derived which determined whether a respondent had a high score (>90% percentile) or not: for women this represented a score ≥14 for depression and ≥10 for anxiety; for men a score ≥10 for depression and ≥7 for anxiety. The women also reported these measures in the same way when their child was aged 5 years. At this time-point, both women and men reported if they had experienced symptoms of anxiety/nerves or depression in the past year.

Social support and networks

During the pregnancy, the participants reported their social support via their level of agreement to ten statements (e.g. 'I believe in moments of difficulty my neighbours would help me'), and their social network via a further ten statements (e.g. 'During the last month, how many times did you get together with one or more friends?'). For both measures, scores were summed with each having a possible range of 0 to 30. A higher score reflects more social support or a better social network. Binary variables were derived to identify those with poor social support or networks, defined as being in the bottom decile. Cut-offs were the same men and women: ≤12 for low social support, ≤18 for poor social network.

Conviction for criminal offence

Participants were asked if they had ever been convicted of a criminal offence in several questionnaires which covered the period from the start of pregnancy, up until the child was aged 5. A binary variable was derived; any conviction (no, yes).

Other variables

Socio-economic position (SEP) were measured during the pregnancy. Household occupational social class was based on the lowest class reported by the woman and her partner (I/II [professional/managerial & technical]; III [skilled manual and skilled non-manual]; IV/V [semi-skilled/unskilled manual] (35)). Men and women also reported their highest educational qualification (university degree; A level; O level; vocational/none). Other measures were reported by women only: financial difficulties (quartiles of score with range 0–40, where 0 is no financial difficulties); housing tenure (owned/mortgaged, private rent, council rent, other); partner status (married; live with partner; do not live with partner; no partner); whether pregnancy with the study child was intentional (no, yes); and their parity (0; 1; 2; 3+). In the 5-year questionnaire, they reported their pregnancy intentions (not pregnant; intend to try later; currently trying; pregnant).

For peer review only

The highest childhood happiness of the men and women was derived from responses to questions on how happy their childhood was at 0-5 years, 6-11 years and 12-15 years (very happy; moderately; quite or very unhappy). School stability was measured by the number of schools attended before the age of 16 years (0-2; 3; 4; 5+). Participants also reported adversities before the age of 17 years: suspended from school; in trouble with police, pregnant (or partner pregnant for the men).

Analysis

The exposure groups were compared in terms of their adult SEP and childhood experiences using descriptive statistics. Logistic regression models were used to examine associations between the exposure and each of the substance use, mental health, social network, and criminal conviction outcomes. Those who had not been adopted or looked-after were the reference group. Models were run unadjusted and adjusted for age and measures related to SEP (relationship status, education, financial difficulties, social class and housing tenure). Models also adjusted for parity for women, and for whether the pregnancy was intentional for the pregnancy time-point outcomes, and for pregnancy intentions for the 5-year time-point outcomes. All analyses were performed stratified by gender.

A sensitivity, cross-sectional analysis of the measures reported during pregnancy only was performed to determine if results were consistent when the sample was not restricted to those who also participated 5 years later.

Missing data

Of the 8775 women in the longitudinal sample, 46.7% had complete data and a further 43.8% had between 1 and 4 missing values. Of the 3654 men, 16.5% had complete data, and a further 78.2% had 1-4 missing values. The percentage of missing data is summarised for each variable in supplementary Table A. Multiple imputation using chained equations was used to replace missing data with predictions based on information observed in the sample. All of the variables included in the analyses models, plus other variables associated with missingness or the variables in the model, were included in the imputation models. Imputed datasets (55 for the women, 100 for the men) were created and analysed using mi estimate commands in Stata 14.2 (StataCorp, College Station, Texas).

In the cross-sectional sample for the sensitivity analysis, complete-case analysis was performed. Of the 11,571 women who reported their childhood care status, 7795 had complete outcome data reported during pregnancy, and of those 7088 had complete confounder data. Of the 7676 men who reported childhood care status, 3163 had complete outcome data reported during their partner’s pregnancy, of whom 2820 had complete confounder data.

3. Results

For peer review only

In the longitudinal sample, 2.4% (95% CI 1.9% to 2.9%) of the men and 2.7% (2.4% to 3.1%) of the women had been adopted. A further 1.4% (1.0% to 1.8%) of the men and 1.8% (1.5% to 2.1%) of the women had been looked-after. These looked-after percentages include the small number of participants who reported that they were unsure if they had been looked-after; excluding them reduced the proportion who were looked-after to 1.1% of men and 1.6% of women.

Of the men who reported their care setting whilst being looked-after, 39% had lived in foster care only, 44% in a children's home only, and 17% in both. Of the women, 30% had lived in foster care, 42% in a children's home, and 28% in both. Over 60% of the men and women who had lived in a children's home had done so for >6 months. Of the adoptees, many had been adopted aged <1 year (women 49%, men 63%). A minority of the adoptees had also been in care (women 14%, men 16%), with a similar proportion reporting that they didn't know.

Compared to the reference group, those who had been looked-after were generally younger, less likely to be married, and of lower SEP (women Table 1; men Table 2). With regards their childhood, they were the least likely to have been very happy, and the most likely to have attended multiple schools, to have been suspended, and in trouble with the police (supplementary Table B). Women who had been looked-after were the most likely to have been pregnant before the age of 17, and to have had several previous pregnancies. They were least likely to report the pregnancy with the study child was intentional, but most likely to be trying to conceive 5 years later. Participants who had been adopted generally had SEP and childhood measures intermediate to those who had been looked-after and those who had not been looked-after or adopted.

Substance Use (Table 3)

Those who had been looked-after or adopted were more likely to be smokers than the reference group at both time-points, with the highest rates observed for looked-after women. Women who had been looked-after or adopted were also more likely to have used cannabis at both time-points. Daily alcohol consumption did not differ between those who had been looked-after and those in the reference group for men or women. Adopted women were the most likely of the women to drink daily pre-pregnancy, but the adopted men were the least likely at both time-points. Rates of addiction to alcohol or drugs were particularly high in men who had been looked-after or adopted. In women, addiction was also more common in those who had been looked-after or adopted, but numbers were small. Adjustment for age, relationship status, and measures of SEP attenuated associations observed for smoking for participants who were looked-after, but had less of an effect on alcohol and cannabis associations, or associations for participants who were adopted.

Mental Health (Table 4)

Women who had been looked-after were the most likely to have high depression and anxiety scores at both time-points, to have ever had a mental health problem, and to have experienced symptoms of anxiety and depression in the past year at the later time-point. Women who had been adopted were also more likely to have high anxiety scores, and to

For peer review only

have had a mental health problem, than those in the reference group. Adjustment attenuated results for the looked-after group but not the adopted group. Men who had been looked-after were more likely to have a high depression score during their partner's pregnancy, and more likely to have ever had a mental health problem, than the reference group. Adjustment for SEP attenuated the differences. At the five-year time-point, there was no difference by care status in the percentage of men reporting symptoms of anxiety or depression in the previous year. Men in each category were less likely to report these symptoms than women, with the gender difference being particularly large for depressive symptoms.

Social support and conviction for criminal offence (Table 5)

Participants who had been looked-after were the most likely to report low social support and a limited social network. Differences relative to the reference group were attenuated on adjustment. Women who had been adopted were more likely to report a poor social network than the reference group, but not a low level of social support. Adopted men did not differ from the reference men.

Men and women who had been looked-after or adopted were more likely to have been convicted of an offence compared to the reference groups. Differences were attenuated on adjustment. Conviction rates were considerably higher for men than women in each of the exposure categories.

Sensitivity analyses

Analyses were repeated excluding the small number of participants who were unsure if they had been looked-after and results were consistent with those of the main analyses. In cross-sectional, complete-case analyses of the outcomes reported in pregnancy only, a slightly higher proportion had been looked-after (men 2.4%; women 1.6%) or adopted (men 3.2%; women 2.6%) compared to the longitudinal sample. The men in the complete case sample were of lower SEP than those in the longitudinal sample (supplementary Table C), but this was not observed for the women (supplementary Table D). Overall, the pattern of results we observed in the longitudinal sample was broadly replicated (supplementary Tables E to G).

Discussion

Overview of findings

We used a population-based study to examine substance use, mental health, social support, and criminal conviction in adulthood amongst individuals who had been looked-after or adopted as children. There was a general, but not universal, pattern of these adults reporting more mental health problems, smoking, and criminal convictions, and less social support, than their peers. Overall, the looked-after individuals reported more negative outcomes than those who had been adopted. There were gender differences in some of the associations observed. For example, women who had been looked after had high rates of

For peer review only

anxiety, whereas men who had been looked-after had an excess risk of addiction and criminality. Adjustment for SEP measures often attenuated associations substantially for the looked-after group, but generally had less of an impact for the adopted group. Rather than implying that adult SEP confounds the relationship between childhood care status and adult psychosocial outcomes, this suggests that it in part mediates these effects.

Comparison to previous literature – adults who have been looked-after

In the UK, the poor outcomes for young care leavers have been widely reported, both in academic literature and increasingly in mainstream media (e.g. 36, 37-40). Our study is one of the few to consider outcomes at an older age. Two previous UK studies have used cohort data to examine the impact of being looked-after on outcomes in adulthood: one used the 1970 British Birth Cohort Study (BCS70) to examine outcomes in men and women aged 30 (41), the other used the Millennium Cohort Study (MCS) to examine outcomes in women mostly aged 20-39 (42). The high rate of depressive symptoms in adults who had been looked after as children in our sample was in concordance with both these studies. Neither included measures of anxiety with which to corroborate our finding of high anxiety in looked-after women but not men.

Whilst the looked-after men reported more addiction, it was for the looked-after women that we found the strongest association with cannabis use. In a qualitative study of care leavers in England, cannabis was viewed as 'relatively harmless', 'acceptable', and 'to have little impact on parenting': some women said it helped them cope with the stress of looking after a baby (12). This could explain our finding of a similar prevalence of cannabis use pre and post pregnancy, in contrast to the decline observed for smoking. Less than 10% of the looked-after individuals in ALSPAC reported cannabis use, a considerably lower percentage than observed in the young care leavers, and in agreement with their general finding that substance use declines with age. However, much higher levels of illegal substance use were reported by the men in the BCS70 (41), suggesting drug use may be low in our sample.

Smoking rates were very high for looked-after men and women. Findings were consistent with previous studies: of the looked-after women in the MCS, 73% had ever been smokers and 58% smoked during pregnancy (42); in the care leavers study, two thirds were daily smokers (12). In contrast, we did not find daily alcohol consumption to be associated with looked-after status in ALSPAC. Our measure does not capture binge drinking behaviours, and reverse causation could be an issue whereby those with alcohol problems had stopped drinking. Results from other studies are inconsistent: in the BCS70 adults who had been looked-after were no more likely to have problems with alcohol (41), but in a Swedish study they were at increased risk of alcohol abuse (17).

The looked-after men and women in ALSPAC were the most likely to report a poor social network and limited social support. The instability of life in care can make it difficult to build and maintain friendships and a support network, and young people can reach adulthood with no family, and no social base (10). This reduced social capital exacerbates their difficulties in transitioning to a successful, independent adult life. For example, their

For peer review only

lack of family support can make accessing and succeeding in post-secondary education difficult (43). Our results show that reduced social capital can persist for many years.

The majority of looked-after children in the UK do not receive a criminal conviction, but as a group they are over-represented in the criminal justice system (26). In our sample, almost a quarter of the looked-after men had been convicted of an offence in the five year period considered. Rates for looked-after women, although lower than the men, were also elevated. Our results highlight that for men in particular, criminal involvement continues for a substantial minority of those who were looked-after, even once they have started a family of their own. Findings were consistent in the BCS70 (41) and the Swedish study (17).

Comparison to previous literature – adults who have been adopted

Comparing outcomes in those adopted versus those who remain in the care system, and identifying causal processes, is complicated because those who successfully complete the adoption process may be systematically different from those who remain in long-term care, and these differences may influence their outcomes in addition to any independent influence of the model of care they receive. The adult SEP of the adoptees in ALSPAC was intermediate to that of the looked-after and reference groups, consistent with the findings of a Swedish study which compared adults aged 25 in the general population to those who had been adopted or fostered (16). In contrast, adopted women in the 1958 British birth cohort had a higher SEP than the general population at age 33, but this was not true for the men (44).

For substance use, no clear pattern emerged when comparing those who had been adopted to those who had been in care. Smoking prevalence and cannabis use were similar for the men, and addiction was similar for the women. Of all the groups, daily alcohol consumption was consistently lowest in the adopted men, whereas of the women those who had been adopted were the most frequent drinkers pre-pregnancy. For men and women in the British 1958 cohort, alcohol problems did not differ between adoptees and the general population (44). Another study used the 1958 cohort data, and similarly found no difference in alcohol abstinence, but the adoptees were more likely to have smoked than the general population. This study also included an additional comparison group, adults who had been adopted from Hong Kong orphanages by British parents; this group were the least likely to drink alcohol or smoke (45). In a Swedish twin study, which included twin pairs aged 40-80 where one twin had been adopted, the adoptees were less likely to report excessive drinking, and this was largely explained by greater family control and higher SEP in their childhood (46).

Adoptees were more likely to report having ever had a mental health problem than the reference group, but less likely than the looked-after group; in contrast the adopted men and women in the 1958 birth cohort did not report excess past emotional problems (44). Adopted women in ALSPAC had high anxiety scores at both time-points relative to the reference group, but again this was not as high as that of the looked-after women. This pattern was not observed for men, and neither gender had excess risk of depression.

For peer review only - http://bmjopen.bmj.com/site/about/guidelines.xhtml

Similarly, in the 1958 cohort the adoptees did not have a higher risk of current depressive symptoms (44) or other mental health problems (45).

Adopted men and women in our study were not more likely than the reference group to have low social support, but the adopted women were more likely to have a limited social network albeit not to the extent observed for the looked-after women. In the 1958 British cohort there was a gender difference: adopted women had the highest level of social support, but adopted men the lowest (44).

Role of early childhood adversity

Many looked-after children have experienced adverse childhood experiences (ACEs), which are associated with poor long term outcomes irrespective of whether the children experiencing these adversities are subsequently looked after (47). For many children being looked-after or adopted is likely to be beneficial. For example, educational attainment improves once they are in long-term care (14). The generally worse outcomes we have found in adults who have been in care should not be interpreted as strong evidence of adverse effects of care itself. Rather, having been in care is a marker of substantial early childhood disadvantage. A recent paper which examined ACEs in the ALSPAC women, without considering childhood care status, found higher levels of mental illness and smoking, and poorer education and social support, in those who had experienced maltreatment in childhood (48). The results mirror ours, and provide evidence for ACEs being important factors in the associations we observe. We were unable to directly examine the role of ACEs in our study as it is not possible to determine if the measures relate to a time before or after a child entered the care system. It is important to acknowledge that becoming looked-after is not inevitably positive for children. Some aspects of care, for example experiences of abuse or neglect within the care system, may compound the effects of early childhood adversity (49).

Limitations

ALSPAC does not include adults who are not parents. To be eligible, women needed to be pregnant, and men needed to be invited by their pregnant partner to participate. We would therefore expect the associations we found to be under-estimates of those in the wider looked-after community, if we assume that our sample have relatively settled lives due to the selection criteria. The participants were children mostly in the 1960s and 70s, and care procedures have changed over time, notably in the greater use of foster as opposed to group care (2), and extended support until age 25 (50). Furthermore, whereas in the past many adoptions were of relinquished babies, now most are of children aged 1-4 years who are in care due to neglect or abuse (51). Therefore our results may not be generalisable to those adopted in the last three decades. The ALSPAC measures of care and adoption lack detail: we do not know the age participants entered care or why, and some of the adoptions may have been by step-parents, rather than as a result of being removed from birth parents. As only a small percentage of children experience care or adoption, numbers in a population-based cohort, even one as large as ALSPAC, will be small.

Implications for practice and policy

For peer review only - http://bmjopen.bmj.com/site/about/guidelines.xhtml

The UK Government has acknowledged the poor life chances of care leavers. The former Prime Minister David Cameron stated in 2015 “we, the state, are their parents – and what are we setting them up for…the dole, the streets, an early grave?” (52). Recent legislative changes have prioritised permanence in the placement of looked-after children, the expedition of adoption of children from care where appropriate, and the provision of personal advisors to care leavers until age 25 (53, 54). For some children, particularly those entering care at an older age, permanence may be best provided through a stable, long-term foster placement. Our results highlight that the wide-ranging needs of those in the care system continue into adulthood, and to when they have a child of their own. They also show that adopted individuals have excess risk in some areas. Long-term, continued improvements to the care system are needed to maximise the life chances of future children who experience adversity in childhood and are not able to grow up with their birth parents. In particular, evidence is needed on which modifiable aspects of care, beyond permanence and placement stability, promote better outcomes. In the meantime, it must be recognised that some of today’s adults who experienced the care system as children have higher needs than those in the general population. In particular, our results suggest a particular need for mental health care, social and educational support, and for services to reduce the harms associated with substance use.

For peer review only

References

1. HM Government. Working together to safeguard children. A guide to inter-agency working to safeguard and promote the welfare of children. 2015 [Available from: www.gov.uk/government/uploads/system/uploads/attachment_data/file/592101/Working_Together_to_Safeguard_Children_20170213.pdf.
2. Department for Education. Children looked after in England (including adoption) year ending 31 March 2016.2016; (Accessed May 2017). Available from: https://www.gov.uk/government/uploads/system/uploads/attachment_data/file/556331/SFR41_2016_Text.pdf.
3. Mc Grath-Lone L, Dearden L, Nasim B, Harron K, Gilbert R. Changes in first entry to out-of-home care from 1992 to 2012 among children in England. Child Abuse Negl. 2016;51:163-71.
4. Mc Grath-Lone L, Harron K, Dearden L, Nasim B, Gilbert R. Data Resource Profile: Children Looked After Return (CLA). International Journal of Epidemiology. 2016;45(3):716-7f.
5. Rubin DM, O'Reilly ALR, Luan X, Localio AR. The Impact of Placement Stability on Behavioral Well-being for Children in Foster Care. Pediatrics. 2007;119(2):336-44.
6. Rock S, Michelson D, Thomson S, Day C. Understanding Foster Placement Instability for Looked After Children: A Systematic Review and Narrative Synthesis of Quantitative and Qualitative Evidence. The British Journal of Social Work. 2015;45(1):177-203.
7. Luke N, Sinclair I, O'Higgins A. The Educational Progress of Looked After Children in England. Technical report 2: Relating Care to Educational Attainment and Progress 2015 [Available from: https://weblearn.ox.ac.uk/access/content/group/5cacf183-ce54-47f0-9f17-d4b0fe1cf292/EducationalProgressLookedAfterChildrenTechnical%20Report%202_Nov2015.pdf.
8. Silver M, Golding K, Roberts C. Paper 9: Delivering psychosocial services for children, young people and families with complex social care needs. 2015 [cited Accessed May 2017]. In: The Child and Family Clinical Psychology Review [Internet]. The British Psychological Society, [cited Accessed May 2017]. Available from: https://shop.bps.org.uk/publications/child-and-family-clinical-psychology-review-no-3-summer-2015.html.
9. Department for Education. Adoption: A Vision for Change 2016 [Available from: https://www.gov.uk/government/uploads/system/uploads/attachment_data/file/512826/Adoption_Policy_Paper_30_March_2016.pdf.
10. Triseliotis J. Long-term foster care or adoption? The evidence examined. Child & Family Social Work. 2002;7(1):23-33.
11. GOV.UK. Leaving foster or local authority care [Available from: www.gov.uk/leaving-foster-or-local-authority-care.
12. Ward J, Henderson Z, Pearson G. One problem among many: drug use among care leavers in transition to independent living. 2003.
13. Department for Education, National Statistics. Outcomes for Children Looked After by Local Authorities in England, as at 31 March 2016. 2017 [Available from: https://www.gov.uk/government/uploads/system/uploads/attachment_data/file/602087/SFR12_2017_Text.pdf.
14. Fletcher J, Strand S, Thomas S. The Educational Progress of Looked After Children in England.

Technical Report 1: Secondary School Progress and Attainment 2015 [Available from: http://reescentre.education.ox.ac.uk/wordpress/wp-content/uploads/2015/11/EducationalProgressLookedAfterChildrenTechnical-Report-1_Nov2015.pdf.
15. The Scottish Government. Education Outcomes for Looked After Children 2014/15 2015 [Available from: http://www.gov.scot/Resource/0050/00501939.pdf.
16. Vinnerljung B, Hjern A. Cognitive, educational and self-support outcomes of long-term foster care versus adoption. A Swedish national cohort study. Children and Youth Services Review. 2011;33(10):1902-10.

17. Berlin M, Vinnerljung B, Hjern A. School performance in primary school and psychosocial problems in young adulthood among care leavers from long term foster care. Children and Youth Services Review. 2011;33(12):2489-97.
18. O'Higgins A, Sebba J, Luke N. What is the relationship between being in care and the educational outcomes of children? An international systematic review 2015 [Available from: http://reescentre.education.ox.ac.uk/wordpress/wp-content/uploads/2015/09/ReesCentreReview_EducationalOutcomes.pdf.
19. FORD T, VOSTANIS P, MELTZER H, GOODMAN R. Psychiatric disorder among British children looked after by local authorities: comparison with children living in private households. The British Journal of Psychiatry. 2007;190(4):319-25.
20. Memarzia J, St Clair MC, Owens M, Goodyer IM, Dunn VJ. Adolescents leaving mental health or social care services: predictors of mental health and psychosocial outcomes one year later. BMC Health Services Research. 2015;15(1):185.
21. Beagley E, Hann G, Al-Bustani N. G156(P) Mental Health Needs of Looked After Children in One of the Most Deprived Boroughs in England. Archives of Disease in Childhood. 2014;99(Suppl 1):A69-A.
22. Lauren Vanderwerker, Ayse Akincigil, Mark Olfson, Tobias Gerhard, Sheree Neese-Todd, Stephen Crystal. Foster Care, Externalizing Disorders, and Antipsychotic Use Among Medicaid-Enrolled Youths. Psychiatric Services. 2014;65(10):1281-4.
23. Pilowsky DJ, Wu L-T. Psychiatric symptoms and substance use disorders in a nationally representative sample of American adolescents involved with foster care. Journal of Adolescent Health. 2006;38(4):351-8.
24. Oswald SH, Heil K, Goldbeck L. History of Maltreatment and Mental Health Problems in Foster Children: A Review of the Literature. Journal of Pediatric Psychology. 2010;35(5):462-72.
25. Tarren-Sweeney M. The mental health of children in out-of-home care. Current opinion in psychiatry. 2008;21(4):345-9.
26. Prison Reform Trust. In Care, Out of Trouble 2016. Available from: http://www.prisonreformtrust.org.uk/Portals/0/Documents/In%20care%20out%20of%20trouble%20summary.pdf.
27. Schofield G, Biggart L, Ward E, Larsson B. Looked after children and offending: An exploration of risk, resilience and the role of social cognition. Children and Youth Services Review. 2015;51:125-33.
28. Brodzinsky DM. Long-Term Outcomes in Adoption. The Future of Children. 1993;3(1):153-66.
29. Wijedasa D, Selwyn J. Transition to adulthood for young people in adoptive care: Secondary analysis of data from the longitudinal study of young people in England (LYSPE). Hadley Centre for Adoption and Foster Care Studies; 2011 [Available from: www.bristol.ac.uk/media-library/sites/sps/migrated/documents/transitiontoadulthood.pdf.
30. Fraser A, Macdonald-Wallis C, Tilling K, Boyd A, Golding J, Smith GD, et al. Cohort profile: the Avon Longitudinal Study of Parents and Children: ALSPAC mothers cohort. International journal of epidemiology. 2013;42(1):97-110.
31. Boyd A, Golding J, Macleod J, Lawlor DA, Fraser A, Henderson J, et al. Cohort profile: the 'children of the 90s'—the index offspring of the Avon Longitudinal Study of Parents and Children. International journal of epidemiology. 2012:dys064.
32. NHS Choices. Alcohol Units 2015 [Available from: http://www.nhs.uk/Livewell/alcohol/Pages/alcohol-units.aspx.
33. Thorpe K. A study of the use of the Edinburgh Postnatal Depression Scale with parent groups outside the postpartum period. Journal of Reproductive and Infant Psychology. 1993;11(2):119-25.
34. Birtchnell J, Evans C, Kennard J. The total score of the Crown-Crisp Experiential Index: A useful and valid measure of psychoneurotic pathology. British Journal of Medical Psychology. 1988;61(3):255-66.

For peer review only

35. Office for Population Censuses & Surveys. Standard Occupational Classification. London; 1991.
36. John-Baptiste A. Leaving care: The financial reality: BBC News; 2017 [Available from: http://www.bbc.co.uk/news/uk-39455547.
37. Greenwood G. Early deaths among care leavers revealed BBC News2017 [Available from: http://www.bbc.co.uk/news/uk-38961818.
38. Slawson N. Girls in state care and care leavers still at high risk of teenage pregnancy. The Guardian. 2016.
39. BBC. Kicked Out: From Care to Chaos 2017 [Available from: http://www.bbc.co.uk/programmes/p04xwl4l.
40. BBC Radio 4. Abandoned to their fate [Internet]; 2015 7 June 2015. Podcast. Available from: http://www.bbc.co.uk/programmes/b05wy64q
41. Viner RM, Taylor B. Adult Health and Social Outcomes of Children Who Have Been in Public Care: Population-Based Study. Pediatrics. 2005;115(4):894-9.
42. Botchway SK, Quigley MA, Gray R. Pregnancy-associated outcomes in women who spent some of their childhood looked after by local authorities: findings from the UK Millennium Cohort Study. BMJ Open. 2014;4(12).
43. Salazar AM, Jones KR, Emerson JC, Mucha L. Postsecondary Strengths, Challenges, and Supports Experienced by Foster Care Alumni College Graduates. Journal of College Student Development. 2016;57(3):263-79.
44. Collishaw S, Maughan B, Pickles A. Infant adoption: psychosocial outcomes in adulthood. Social Psychiatry and Psychiatric Epidemiology. 1998;33(2):57-65.
45. Rushton A, Grant M, Feast J, Simmonds J. The British Chinese Adoption Study: orphanage care, adoption and mid-life outcomes. Journal of Child Psychology and Psychiatry. 2013;54(11):1215-22.
46. Smyer MA, Gatz M, Simi NL, Pedersen NL. Childhood adoption: Long-term effects in adulthood. Psychiatry. 1998;61(3):191-205.
47. Bellis MA, Lowey H, Leckenby N, Hughes K, Harrison D. Adverse childhood experiences: retrospective study to determine their impact on adult health behaviours and health outcomes in a UK population. Journal of Public Health. 2014;36(1):81-91.
48. Plant DT, Jones FW, Pariante CM, Pawlby S. Association between maternal childhood trauma and offspring childhood psychopathology: mediation analysis from the ALSPAC cohort. The British Journal of Psychiatry. 2017.
49. Biehal N, Cusworth L, Wade J, Clarke S. Keeping children safe: allegations concerning the abuse or neglect of children in care. University of York: NSPCC; 2014 [Available from: www.nspcc.org.uk/globalassets/documents/evaluation-of-services/keeping-children-safe-report.pdf.
50. UK Government. Children (Leaving Care) Act 2000 2000 [Available from: http://www.legislation.gov.uk/ukpga/2000/35/contents.
51. Adoption UK. Adoption facts and figures for England 2017 [Available from: www.adoptionuk.org/press-media/adoption-facts-and-figures-england.
52. Cameron D. Prime Minister: Conference speech 2015 2015 [Available from: http://press.conservatives.com/post/130746609060/prime-minister-conference-speech-2015.
53. UK Government. Children and Social Work Act: The Stationary Office; 2017 [Available from: http://www.legislation.gov.uk/ukpga/2017/16/pdfs/ukpga_20170016_en.pdf.
54. GOV.UK. Education Secretary unveils plans to change adoption law 2016 [Available from: https://www.gov.uk/government/news/education-secretary-unveils-plans-to-change-adoption-law.

For peer review only

Footnotes

Contributors: AT conceived the study, conducted the analyses, interpreted the data, and drafted the manuscript. JM helped develop the research question, interpreted the data, and critically revised the paper. DW interpreted the data and critically revised the paper. All authors have read and approved this final version. AT will serve as guarantor for the contents of this paper.

Competing interests JM is a foster carer. AT and DW have no competing interests.

Funding The UK Medical Research Council and the Wellcome Trust (Grant Ref: 092731) and the University of Bristol provide core support for ALSPAC. AT and JM are supported by PEARL (Project to Enhance ALSPAC through Record Linkage), a programme of research funded by the Wellcome Trust (WT086118/Z/08/Z), and The Farr Institute CIPHER, which is supported by a 10-funder consortium: Arthritis Research UK, the British Heart Foundation, Cancer Research UK, the Economic and Social Research Council, the Engineering and Physical Sciences Research Council, the Medical Research Council, the National Institute of Health Research, the National Institute for Social Care and Health Research (Welsh Assembly Government), the Chief Scientist Office (Scottish Government Health Directorates), and the Wellcome Trust (MRC Grant No: MR/K006525/1).

Acknowledgements We are grateful to all the families who took part in ALSPAC, the midwives for their help in recruiting them, and the ALSPAC team, which includes interviewers, computer and laboratory scientists, clerical workers, research scientists, volunteers, managers, receptionists, and nurses.

Ethics approval Ethical approval for ALSPAC was obtained from the ALSPAC Ethics and Law Committee and the Local Research Ethics Committees (LREC). Full LREC details are available online (http://www.bristol.ac.uk/alspac/researchers/research-ethics/). This study was approved by the ALSPAC Executive Committee. It involves secondary analysis of ALSPAC questionnaire data, which is fully anonymised before researchers can access or analyse it.

Data sharing statement The ALSPAC data management plan (available here: http://www.bristol.ac.uk/alspac/researchers/data-access/ documents/alspac-data-management-plan.pdf) describes in detail the policy regarding data sharing, which is through a system of managed open access. Requests for the data used in this paper can be made to the Executive (alspac-exec@bristol.ac.uk).

For peer review only

Table 1: Characteristics of the women by care status in childhood, n=8775

		Reference group (95.5%)	Looked After (1.8%)	Adopted (2.7%)
Age at delivery	Mean age in years	28.8 (28.7-28.9)	27.6 (26.6-28.5)	27.7 (27.2-28.3)
	<=23 years (%)	11.9 (11.2-12.6)	26.7 (19.4-34.1)	15.2 (10.3-20.2)
	>=34 years (%)	15.4 (14.6-16.2)	15.4 (9.3-21.4)	8.7 (5.0-12.5)
Relationship status	Married (%)	80.3 (79.4-81.2)	59.8 (51.4-68.2)	78.3 (72.8-83.8)
	Resident partner (%)	13.4 (12.7-14.2)	20.4 (13.3-27.5)	12.6 (8.1-17.1)
	Non-resident partner (%)	4.4 (4.0-4.9)	14.0 (8.0-20.0)	7.0 (3.5-10.5)
	No partner (%)	1.9 (1.6-2.2)	5.8 (1.7-9.9)	2.1 (0-4.1)
Parity	0 (%)	46.0 (45.0-47.1)	36.3 (28.0-44.7)	48.3 (41.7-54.9)
	3+ (%)	4.6 (4.1-5.1)	15.5 (9.3-21.6)	5.3 (2.4-8.2)
This pregnancy intentional	Yes (%)	73.3 (72.3-74.3)	53.5 (45.0-61.9)	69.2 (63.1-75.4)
Pregnancy intentions at 5 years	Pregnant (%)	3.8 (3.4-4.2)	3.3 (2.5-11.3)	4.8 (1.9-7.8)
	Trying to get pregnant (%)	2.2 (1.9-2.5)	6.9 (2.5-11.3)	1.9 (0-3.7)
Highest maternal education	Degree (%)	15.2 (14.4-16.0)	2.7 (0-5.5)	11.2 (7.0-15.4)
	Vocational/none (%)	24.3 (23.3-25.2)	48.8 (39.8-57.8)	21.8 (16.1-27.4)
Financial difficulties	Q1 (none) (%)	39.4 (38.3-40.4)	22.8 (15.7-29.8)	29.8 (23.6-35.9)
	Q4 (high) (%)	17.7 (16.8-18.5)	33.2 (24.8-41.6)	20.1 (14.7-25.5)
Housing tenure	Owned/mortgaged (%)	80.6 (79.8-81.5)	48.1 (39.6-56.6)	74.8 (69.0-80.6)
Lowest social class of self and partner	I&II (%)	27.9 (26.9-28.9)	16.7 (10.1-23.3)	25.5 (19.6-31.4)
	IV&V (%)	18.1 (17.2-18.9)	26.1 (17.5-34.7)	21.3 (15.6-27.1)

For peer review only

Table 2: Characteristics of the men by care status in childhood, n=3654

		Reference Group (96.1%)	Looked After (1.4%)	Adopted (2.4%)
Age when partner at 18wks gestation	Mean (years)	31.5 (31.3-31.7)	30.5 (29.1-32.0)	30.4 (29.1-31.8)
	<=23 years (%)	3.4 (2.8-4.1)	4.7 (0-11.2)	7.7 (1.7-13.6)
	>=34 years (%)	30.2 (28.6-31.7)	26.8 (13.8-39.9)	23.5 (14.3-32.7)
Relationship status	Married (%)	88.1 (87.0-89.2)	78.8 (67.4-90.3)	78.5 (69.7-87.2)
	Resident partner (%)	10.3 (9.3-11.3)	21.2 (9.7-32.6)	20.4 (11.8-28.9)
	Non-resident partner (%)	1.6 (1.2-2.0)	0	1.2 (0-3.4)
Highest education	Degree (%)	29.3 (27.7-30.8)	4.5 (0-10.8)	18.6 (10.2-27.0)
	Vocational/none (%)	19.3 (17.9-20.6)	53.3 (38.8-67.8)	18.3 (9.9-26.7)
Financial difficulties	Q1 (none) (%)	47.1 (45.5-48.8)	21.9 (10.1-33.7)	33.5 (23.2-43.7)
	Q4 (high) (%)	12.5 (11.4-13.7)	29.2 (16.3-42.1)	19.3 (10.5-28.1)
Housing tenure	Owned/mortgaged (%)	87.4 (86.3-88.5)	53.8 (39.8-67.9)	75.0 (65.8-84.3)
Lowest social class of self and partner	I&II (%)	35.8 (34.2-37.4)	13.8 (4.0-23.7)	21.7 (12.6-30.7)
	IV&V (%)	14.9 (13.7-16.1)	24.9 (12.1-37.7)	17.8 (9.1-26.6)

For peer review only

Table 3: Substance use outcomes by childhood care status for men and women

		Men			Women		
		%	OR (95% CI)		%	OR (95% CI)	
			Unadjusted	Adjusted[1]		Unadjusted	Adjusted[2]
Has ever had addiction	Reference	2.0	Ref	Ref	1.0	Ref	Ref
	Looked After	13.5	7.8 (3.4-17.8)*	4.0 (1.6-10.0)*	3.1	2.9 (0.9-9.1)	1.6 (0.5-5.4)
	Adopted	6.8	3.6 (1.5-8.6)*	2.3 (0.9-6.0)	3.0	2.9 (1.3-6.8)*	2.8 (1.2-6.6)*
**Pre-pregnancy**							
Smoked regularly	Reference	28.0	Ref	Ref	28.3	Ref	Ref
	Looked After	46.5	2.2 (1.3-3.9)*	1.2 (0.7-2.2)	57.9	3.5 (2.5-4.9)*	1.9 (1.3-2.8)*
	Adopted	44.3	2.0 (1.3-3.2)*	1.7 (1.1-2.7)*	40.3	1.7 (1.3-2.3)*	1.6 (1.2-2.2)*
Drank alcohol daily	Reference	23.8	Ref	Ref	11.3	Ref	Ref
	Looked After	21.8	0.9 (0.5-1.7)	1.1 (0.6-2.3)	9.6	0.8 (0.4-1.5)	0.9 (0.5-1.7)
	Adopted	11.4	0.4 (0.2-0.8)*	0.4 (0.2-0.8)*	17.0	1.6 (1.1-2.3)*	1.8 (1.3-2.6)*
Used cannabis	Reference	6.6	Ref	Ref	3.9	Ref	Ref
	Looked After	9.1	1.3 (0.4-4.7)	1.0 (0.3-3.6)	11.2	3.1 (1.8-5.5)*	2.3 (1.2-4.3)*
	Adopted	9.9	1.5 (0.7-3.4)	1.1 (0.5-2.7)	5.5	1.4 (0.8-2.6)	1.2 (0.6-2.2)
**When study child 5 years**							
Smoked regularly	Reference	22.3	Ref	Ref	23.1	Ref	Ref
	Looked After	44.2	2.8 (1.6-4.8)*	1.6 (0.9-2.9)	52.9	3.7 (2.7-5.3)*	2.0 (1.4-3.0)*
	Adopted	40.3	2.4 (1.5-3.6)*	2.0 (1.2-3.2)*	32.5	1.6 (1.2-2.1)*	1.5 (1.1-2.1)*
Drank alcohol daily	Reference	35.9	Ref	Ref	16.4	Ref	Ref
	Looked After	26.9	0.7 (0.4-1.3)	1.1 (0.6-2.0)	11.0	0.6 (0.4-1.1)	0.9 (0.5-1.6)
	Adopted	19.4	0.4 (0.3-0.8)*	0.5 (0.3-0.9)*	17.0	1.0 (0.7-1.5)	1.2 (0.8-1.7)
Used cannabis	Reference	6.1	Ref	Ref	4.2	Ref	Ref
	Looked After	8.5	1.4 (0.5-3.9)	1.0 (0.3-2.9)	8.8	2.2 (1.2-4.1)*	1.5 (0.7-2.9)
	Adopted	9.9	1.7 (0.8-3.5)	1.2 (0.6-2.6)	8.5	2.1 (1.3-3.5)*	1.9 (1.1-3.1)*

For peer review only

[1]Adjusted for age, relationship status, education, financial difficulties, social class, housing tenure. [2]Adjusted for age, relationship status, education, financial difficulties, social class, housing tenure, parity, pregnancy intentional (pre-pregnancy models only), pregnancy status/intentions (5 year models only). *$p<0.05$

For peer review only

Table 4: Mental health outcomes by childhood care status for men and women

		Men			Women		
		%	**OR (95% CI)**[1]		%	**OR (95% CI)**[2]	
			Unadjusted	Adjusted[1]		Unadjusted	Adjusted[1]
Has ever had mental health problem	Reference	5.7	Ref	Ref	10.2	Ref	Ref
	Looked After	24.1	5.3 (2.7-10.2)*	3.9 (1.9-7.8)*	25.9	3.1 (2.1-4.5)*	2.2 (1.4-3.2)*
	Adopted	12.4	2.4 (1.2-4.5)*	2.0 (1.0-3.9)	15.5	1.6 (1.1-2.3)*	1.6 (1.1-2.3)*
**During pregnancy**							
High anxiety score (>6m, >9w)	Reference	9.3	Ref	Ref	10.4	Ref	Ref
	Looked After	15.2	1.7 (0.8-3.9)	1.6 (0.7-3.7)	26.1	3.0 (2.0-4.5)*	2.0 (1.3-3.1)*
	Adopted	11.1	1.2 (0.6-2.4)	1.2 (0.6-2.4)	17.4	1.8 (1.2-2.6)*	1.6 (1.1-2.4)*
High depression score (>8m, >13w)	Reference	11.0	Ref	Ref	8.7	Ref	Ref
	Looked After	26.2	2.9 (1.5-5.6)*	2.3 (1.1-4.5)*	24.3	3.4 (2.2-5.0)*	2.0 (1.3-3.1)*
	Adopted	12.3	1.1 (0.6-2.2)	1.0 (0.5-1.9)	10.9	1.3 (0.8-2.0)	1.1 (0.7-1.8)
**When study child 5 years**							
High anxiety score[2]	Reference	/	/	/	10.1	Ref	Ref
	Looked After	/	/	/	18.7	2.0 (1.3-3.2)*	1.5 (1.0-2.5)
	Adopted	/	/	/	14.3	1.5 (1.0-2.2)*	1.4 (1.0-2.1)
High depression score[2]	Reference	/	/	/	9.5	Ref	Ref
	Looked After	/	/	/	18.5	2.2 (1.4-3.4)*	1.6 (1.0-2.5)*
	Adopted	/	/	/	12.2	1.3 (0.9-2.0)	1.3 (0.8-1.9)
Anxiety symptoms in past year	Reference	18.4	Ref	Ref	22.5	Ref	Ref
	Looked After	17.1	0.9 (0.4-1.9)	1.0 (0.5-2.2)	31.4	1.6 (1.1-2.3)*	1.4 (1.0-2.1)
	Adopted	17.8	1.0 (0.5-1.7)	1.0 (0.5-1.7)	25.8	1.2 (0.9-1.6)	1.2 (0.9-1.6)
Depression symptoms in past year	Reference	12.6	Ref	Ref	23.0	Ref	Ref
	Looked After	15.0	1.2 (0.6-2.7)	1.0 (0.4-2.2)	38.8	2.1 (1.5-3.0)*	1.6 (1.1-2.3)*
	Adopted	8.2	0.6 (0.3-1.4)	0.5 (0.2-1.2)	27.3	1.3 (0.9-1.7)	1.2 (0.9-1.6)

[1]Adjusted for age, relationship status, education, financial difficulties, social class, housing tenure. [2]Adjusted for age, relationship status, education, financial difficulties, social class, housing tenure, parity, pregnancy intentional (pregnancy models only), pregnancy status/intentions (5 year models only). *p<0.05

For peer review only

Table 5: Social support and criminal involvement outcomes by childhood care status for men and women

		Men			Women		
		**%**	**OR (95% CI)[1]**		**%**	**OR (95% CI)[2]**	
			Unadjusted	Adjusted[1]		Unadjusted	Adjusted[1]
**During pregnancy**							
Low social support	Reference	10.8	Ref	Ref	8.1	Ref	Ref
	Looked After	25.2	2.8 (1.4-5.4)*	2.2 (1.1-4.4)*	22.5	3.3 (2.1-5.0)*	1.6 (1.0-2.5)
	Adopted	8.7	0.8 (0.4-1.7)	0.7 (0.3-1.5)	10.5	1.3 (0.8-2.1)	1.2 (0.7-2.0)
Poor social network	Reference	12.7	Ref	Ref	8.8	Ref	Ref
	Looked After	26.4	2.5 (1.3-4.9)*	1.9 (1.0-3.8)	26.3	3.7 (2.5-5.5)*	2.0 (1.3-3.1)*
	Adopted	14.1	1.2 (0.6-2.1)	1.1 (0.6-2.1)	13.2	1.6 (1.0-2.3)*	1.5 (1.0-2.3)
**From pregnancy to child aged 5**							
Any criminal conviction	Reference	6.9	Ref	Ref	1.4	Ref	Ref
	Looked After	23.1	3.9 (1.5-10.4)*	2.6 (0.9-7.5)	6.2	4.4 (1.7-11.5)*	2.4 (0.9-6.5)
	Adopted	15.0	2.3 (1.1-5.0)*	1.8 (0.8-4.2)	4.6	3.2 (1.4-7.5)*	2.9 (1.2-7.1)*

[1]Adjusted for age, relationship status, education, financial difficulties, social class, housing tenure.

[2]Adjusted for age, relationship status, education, financial difficulties, social class, housing tenure, parity, pregnancy intentional (pregnancy models only), pregnancy status/intentions (5 year models only). *p<0.05

For peer review only

Supplementary Tables

Table A: Summary of missing data

Table B: Summary of childhood and adolescent measures by care status for women and men

Tables C-G: Results for sensitivity analyses (complete case, pregnancy time-point outcomes only)

For peer review only

Table A: Summary of missing data

		N (%) missing	
		**Women (100%=8775)**	**Men (100%=3654)**
**Exposure variables**	Looked after status	588 (6.7)	0
	Adoption status	313 (3.6)	13 (0.4)
**Outcomes**			
(a) Pre-pregnancy (or pre-partner's pregnancy)	**Substance use**		
	Alcohol - frequency	182 (2.1)	99 (2.7)
	Smoking	145 (1.7)	142 (3.9)
	Cannabis	504 (5.7)	1771 (48.5)
	Ever had addiction	243 (2.8)	6 (0.2)
(b) Pregnancy (or partner's pregnancy)	**Mental health**		
	Depressive symptoms (EPDS)	799 (9.1)	134 (3.7)
	Anxiety symptoms (Crown-crisp)	884 (10.1)	134 (3.7)
	Ever had mental health problem	243 (2.8)	15 (0.4)
	**Social environment**		
	Social support	795 (9.1)	245 (6.7)
	Social network	422 (4.8)	188 (5.1)
(c) Child aged 5 years	**Substance use**		
	Alcohol - frequency	39 (0.4)	14 (0.4)
	Alcohol - binge	127 (1.5)	N/A
	Smoking	114 (1.3)	93 (2.6)
	Cannabis	52 (0.6)	47 (1.3)
	**Mental health**		
	Depressive symptoms (EPDS)	104 (1.2)	N/A
	Anxiety symptoms (Crown-crisp)	155 (1.8)	N/A
	Anxiety/nerves in past year	184 (2.1)	79 (2.2)
	Depression in past year	171 (1.9)	89 (2.4)
(d) Pregnancy (or partner's pregnancy) to child aged 5 years	Any criminal conviction	1681 (19.2)	1265 (34.6)
**Other**	Age	0	192 (5.3)
	Educational attainment	251 (2.9)	105 (2.9)
	Housing tenure	216 (2.5)	20 (0.6)
	Financial difficulties	481 (5.5)	115 (3.2)
	Social class	721 (8.2)	139 (3.8)
	Partner status	160 (1.8)	2 (0.1)
	Pregnancy with study child intentional	184 (2.1)	21 (0.6)
	Pregnancy intentions at 5 yrs	92 (1.1)	N/A
	Childhood happiness	93 (1.1)	44 (1.2)
	Pregnancy (own or partner's) pre 17yrs	272 (3.1)	88 (2.4)
	Suspended from school pre 17yrs	272 (3.1)	88 (2.4)
	In trouble with police pre 17yrs	272 (3.1)	88 (2.4)
	Number of schools attended	655 (7.5)	900 (24.6)

For peer review only

Table B: Summary of childhood and adolescent measures by care status for women and men

		Women			Men		
		Reference	Looked After	Adopted	Reference	Looked After	Adopted
Highest childhood happiness	Very happy (%)	75.4 (74.4-76.3)	46.9 (38.4-55.3)	72.9 (67.0-78.8)	77.9 (76.5-79.3)	61.9 (48.0-75.8)	77.2 (68.3-86.2)
Number of schools attended by 16 yrs	0-2 (%)	43.3 (42.2-44.4)	16.9 (10.0-23.8)	39.8 (33.0-46.6)	42.0 (40.2-43.8)	20.4 (6.2-34.6)	40.0 (28.1-52.0)
	5+ (%)	7.9 (7.3-8.4)	29.7 (21.6-37.9)	12.5 (8.1-16.9)	8.1 (7.1-9.1)	22.3 (8.2-36.5)	9.3 (2.2-16.4)
Suspended from school pre 17 years	Yes (%)	2.4 (2.1-2.7)	16.1 (9.7-22.4)	6.0 (2.6-9.3)	6.2 (5.4-7.0)	15.2 (4.5-25.9)	12.3 (5.2-19.4)
In trouble with police pre 17 years	Yes (%)	3.2 (2.8-3.6)	14.4 (8.3-20.5)	8.3 (4.4-12.1)	13.5 (12.3-14.6)	41.3 (27.0-55.6)	30.1 (20.3-40.0)
Pregnancy pre 17 years	Yes (%)	5.6 (5.1-6.1)	22.4 (15.2-29.6)	8.3 (4.5-12.2)	1.7 (1.3-2.2)	6.1 (0-13.1)	5.0 (0.2-9.8)

For peer review only

Table C: Characteristics of the women by care status in childhood, n=7088 (complete case, cross-sectional sample)

		Not looked after or adopted N=6794	Looked After (not adopted) N=110	Adopted N=184
Maternal age at delivery	Mean (years)	28.6	27.2	27.7
	<=23 years (%)	12.5 (11.7-13.3)	25.5 (18.1-34.6)	15.8 (11.1-21.8)
	>=34 years (%)	14.1 (13.3-14.9)	10.0 (5.6-17.3)	9.8 (6.2-15.1)
Relationship status	Husband (%)	82.1 (81.2-83.0)	62.7 (53.2-71.4)	78.8 (72.2-84.1)
	Resident partner (%)	13.8 (13.0-14.6)	21.8 (15.0-30.6)	15.2 (10.7-21.2)
	Non-resident partner (%)	3.6 (3.2-4.1)	13.6 (8.3-21.5)	4.9 (2.5-9.2)
	No partner (%)	0.4 (0.3-0.6)	1.8 (0.4-7.1)	1.1 (0.3-4.3)
Parity	0 (%)	44.6 (43.4-45.8)	29.1 (21.3-38.4)	48.9 (41.7-56.2)
	3+ (%)	4.6 (4.1-5.1)	21.0 (14.2-29.7)	7.1 (4.1-11.8)
This pregnancy intentional	Yes (%)	75.1 (74.1-76.2)	53.6 (44.2-62.9)	71.2 (64.2-77.3)
Highest maternal education	Degree (%)	14.0 (13.2-14.9)	3.6 (1.4-9.4)	8.2 (5.0-13.2)
	Vocational/none (%)	23.8 (22.8-24.9)	53.6 (44.2-62.9)	22.8 (17.3-29.5)
Financial difficulties	Q1 (none) (%)	39.0 (37.9-40.2)	17.3 (11.2-25.7)	26.6 (20.7-33.5)
	Q4 (high) (%)	17.4 (16.5-18.3)	36.4 (27.8-45.9)	19.6 (14.4-26.0)
Housing tenure	Owned/mortgaged (%)	81.5 (80.6-82.4)	43.6 (34.6-53.2)	75.5 (68.8-81.3)
Lowest social class of self and partner	I&II	26.5 (25.4-27.5)	17.3 (11.2-25.7)	25.5 (19.7-32.4)
	IV&V	18.9 (18.0-19.8)	26.4 (18.9-35.5)	23.9 (18.3-30.7)

For peer review only

Table D: Characteristics of the men by care status in childhood, n=2820 (complete case, cross-sectional sample)

		Not looked after or adopted N=2662	Looked After (not adopted) N=68	Adopted N=90
Paternal age at 18wks gestation	Mean (years)	30.9	29.6	29.2
	<=23 years (%)	7.6 (6.7-8.7)	16.2 (9.1-27.2)	12.2 (30.3-50.6)
	>=34 years (%)	29.0 (27.3-30.7)	20.6 (12.4-32.2)	20.0 (12.9-29.7)
Relationship status	Husband (%)	81.1 (79.6-82.5)	72.1 (60.0-81.6)	73.3 (63.1-81.6)
	Resident partner (%)	15.8 (14.4-17.2)	25.0 (16.0-36.9)	23.3 (15.6-33.4)
	Non-resident partner (%)	3.1 (2.5-2.9)	2.9 (0.7-11.4)	3.3 (1.1-10.0)
Highest education	Degree (%)	18.2 (16.8-19.7)	2.9 (0.7-11.4)	14.4 (8.5-23.5)
	Vocational/none (%)	28.1 (26.4-29.8)	54.4 (42.2-66.1)	31.1 (22.3-41.6)
Financial difficulties	Q1 (none) (%)	37.0 (35.2-38.9)	14.7 (8.0-25.5)	26.7 (18.4-36.9)
	Q4 (high) (%)	18.8 (17.3-20.3)	44.1 (32.6-56.3)	24.4 (16.5-34.6)
Housing tenure	Owned/mortgaged (%)	78.4 (76.8-80.0)	47.1 (35.3-59.2)	67.8 (57.3-76.8)
Lowest social class of self and partner	I&II	26.3 (24.6-28.0)	17.6 (10.2-28.9)	26.7 (18.4-36.9)
	IV&V	21.3 (19.7-22.9)	32.4 (22.1-44.6)	22.2 (14.7-32.2)

For peer review only

Table E: Substance use pre-pregnancy by childhood care status for men and women (complete case, cross-sectional sample)

		Men			Women		
		%	OR (95% CI)		%	OR (95% CI)	
**Outcome**	**Care status**		Unadjusted	Adjusted[1]		Unadjusted	Adjusted[2]
Smoked regularly	None	57.0	Ref	Ref	29.2	Ref	Ref
	Looked After	76.5	2.5 (1.4-4.3)*	1.4 (0.8-2.6)	61.8	3.9 (2.7-5.8)*	1.9 (1.3-3.0)*
	Adopted	64.4	1.4 (0.9-2.1)	1.2 (0.7-1.8)	41.8	1.7 (1.3-2.3)*	1.6 (1.1-2.2)*
Drank alcohol every day	None	26.7	Ref	Ref	11.1	Ref	Ref
	Looked After	17.6	0.6 (0.3-1.1)	0.8 (0.4-1.5)	7.3	0.6 (0.3-1.3)	0.8 (0.4-1.6)
	Adopted	20.0	0.7 (0.4-1.2)	0.7 (0.4-1.2)	21.2	2.2 (1.5-3.1)*	2.4 (1.7-3.5)*
Used cannabis	None	10.1	Ref	Ref	3.5	Ref	Ref
	Looked After	11.8	1.2 (0.6-2.5)	0.8 (0.3-1.7)	9.1	2.7 (1.4-5.3)*	1.6 (0.8-3.3)
	Adopted	17.8	1.9 (1.1-3.4)*	1.5 (0.8-2.8)	8.2	2.4 (1.4-4.2)*	2.0 (1.1-3.7)*
Has ever had addiction	None	4.3	Ref	Ref	0.8	Ref	Ref
	Looked After	14.7	3.8 (1.9-7.7)*	2.1 (1.0-4.5)	3.6	4.6 (1.6-13.0)*	2.3 (0.8-6.7)
	Adopted	7.8	1.9 (0.8-4.1)	1.6 (0.7-3.5)	2.2	2.7 (1.0-7.6)	2.3 (0.8-6.6)

[1]Adjusted for age, relationship status, education, financial difficulties, social class, housing tenure.

[2]Adjusted for age, relationship status, education, financial difficulties, social class, housing tenure, parity, pregnancy intentional.

*p<0.05

For peer review only

Table F: Mental health outcomes during the pregnancy by childhood care status for men and women (complete case, cross-sectional sample)

		Men			Women		
		%	**OR (95% CI)**		%	**OR (95% CI)**	
			Unadjusted	Adjusted[1]		Unadjusted	Adjusted[2]
High anxiety score (>6m, >9w)	None	10.6	Ref	Ref	10.4	Ref	Ref
	Looked After	14.7	1.5 (0.7-2.9)	1.3 (0.6-2.6)	26.4	3.1 (2.0-4.8)*	1.7 (1.1-2.7)*
	Adopted	11.1	1.1 (0.5-2.1)	1.0 (0.5-2.1)	19.0	2.0 (1.4-3.0)*	1.8 (1.2-2.6)*
High depression score (>8m, >13w)	None	12.7	Ref	Ref	8.6	Ref	Ref
	Looked After	22.1	2.0 (1.1-3.5)*	1.4 (0.8-2.6)	20.9	2.8 (1.8-4.5)*	1.5 (0.9-2.4)
	Adopted	12.2	1.0 (0.5-1.8)	0.9 (0.5-1.7)	16.3	2.1 (1.4-3.1)*	1.8 (1.2-2.8)*
Ever had mental health problem	None	8.3	Ref	Ref	10.0	Ref	Ref
	Looked After	17.6	2.4 (1.3-4.5)*	1.6 (0.8-3.2)	22.7	2.7 (1.7-4.2)*	1.7 (1.0-2.7)*
	Adopted	17.8	2.4 (1.4-4.2)*	2.2 (1.2-3.9)*	17.4	1.9 (1.3-2.8)*	1.8 (1.2-2.7)*

[1]Adjusted for age, relationship status, education, financial difficulties, social class, housing tenure.
[2]Adjusted for age, relationship status, education, financial difficulties, social class, housing tenure, parity, pregnancy intentional.
*p<0.05

For peer review only

Table G: Social support outcomes by childhood care status for men and women (complete case, cross-sectional sample)

		Men			Women		
	**Care status**	**%**	**OR (95% CI)**		**%**	**OR (95% CI)**	
			Unadjusted	Adjusted[1]		Unadjusted	Adjusted[2]
Low social support	None	13.9	Ref	Ref	8.0	Ref	Ref
	Looked After	26.5	2.2 (1.3-3.9)*	1.7 (1.0-3.0)	21.8	3.2 (2.0-5.1)*	1.5 (0.9-2.4)
	Adopted	15.6	1.1 (0.6-2.0)	1.1 (0.6-1.9)	9.8	1.2 (0.8-2.0)	1.1 (0.7-1.8)
Poor social network	None	15.8	Ref	Ref	8.6	Ref	Ref
	Looked After	22.1	1.5 (0.8-2.7)	1.0 (0.5-1.8)	24.5	3.5 (2.2-5.4)*	1.6 (1.0-2.6)*
	Adopted	15.6	1.0 (0.6-1.8)	0.9 (0.5-1.7)	13.6	1.7 (1.1-2.6)*	1.5 (1.0-2.4)

[1]Adjusted for age, relationship status, education, financial difficulties, social class, housing tenure.

[2]Adjusted for age, relationship status, education, financial difficulties, social class, housing tenure, parity, pregnancy intentional.

*p<0.05

For peer review only

STROBE 2007 (v4) Statement—Checklist of items that should be included in reports of *cohort studies*

Section/Topic	Item #	Recommendation	Reported on page #
**Title and abstract**	1	(*a*) Indicate the study's design with a commonly used term in the title or the abstract	1,2
		(*b*) Provide in the abstract an informative and balanced summary of what was done and what was found	2
**Introduction**			
Background/rationale	2	Explain the scientific background and rationale for the investigation being reported	4-5
Objectives	3	State specific objectives, including any prespecified hypotheses	2,4-5
**Methods**			
Study design	4	Present key elements of study design early in the paper	4-5
Setting	5	Describe the setting, locations, and relevant dates, including periods of recruitment, exposure, follow-up, and data collection	5
Participants	6	(*a*) Give the eligibility criteria, and the sources and methods of selection of participants. Describe methods of follow-up	5
		(*b*) For matched studies, give matching criteria and number of exposed and unexposed	N/A
Variables	7	Clearly define all outcomes, exposures, predictors, potential confounders, and effect modifiers. Give diagnostic criteria, if applicable	5-7
Data sources/ measurement	8*	For each variable of interest, give sources of data and details of methods of assessment (measurement). Describe comparability of assessment methods if there is more than one group	5-7
Bias	9	Describe any efforts to address potential sources of bias	7
Study size	10	Explain how the study size was arrived at	5
Quantitative variables	11	Explain how quantitative variables were handled in the analyses. If applicable, describe which groupings were chosen and why	5-7
Statistical methods	12	(*a*) Describe all statistical methods, including those used to control for confounding	7
		(*b*) Describe any methods used to examine subgroups and interactions	N/A
		(*c*) Explain how missing data were addressed	7
		(*d*) If applicable, explain how loss to follow-up was addressed	7
		(*e*) Describe any sensitivity analyses	7
**Results**			

For peer review only

Participants	13*	(a) Report numbers of individuals at each stage of study—eg numbers potentially eligible, examined for eligibility, confirmed eligible, included in the study, completing follow-up, and analysed	5,7
		(b) Give reasons for non-participation at each stage	5
		(c) Consider use of a flow diagram	N/A
Descriptive data	14*	(a) Give characteristics of study participants (eg demographic, clinical, social) and information on exposures and potential confounders	8, Tables 1, 2 and B
		(b) Indicate number of participants with missing data for each variable of interest	Table A
		(c) Summarise follow-up time (eg, average and total amount)	5
Outcome data	15*	Report numbers of outcome events or summary measures over time	8-9
Main results	16	(*a*) Give unadjusted estimates and, if applicable, confounder-adjusted estimates and their precision (eg, 95% confidence interval). Make clear which confounders were adjusted for and why they were included	Tables 3-5
		(*b*) Report category boundaries when continuous variables were categorized	5-7
		(*c*) If relevant, consider translating estimates of relative risk into absolute risk for a meaningful time period	N/A
Other analyses	17	Report other analyses done—eg analyses of subgroups and interactions, and sensitivity analyses	9
**Discussion**			
Key results	18	Summarise key results with reference to study objectives	9-10
**Limitations**			
Interpretation	20	Give a cautious overall interpretation of results considering objectives, limitations, multiplicity of analyses, results from similar studies, and other relevant evidence	10-12
Generalisability	21	Discuss the generalisability (external validity) of the study results	12
**Other information**			
Funding	22	Give the source of funding and the role of the funders for the present study and, if applicable, for the original study on which the present article is based	17

*Give information separately for cases and controls in case-control studies and, if applicable, for exposed and unexposed groups in cohort and cross-sectional studies.

Note: An Explanation and Elaboration article discusses each checklist item and gives methodological background and published examples of transparent reporting. The STROBE checklist is best used in conjunction with this article (freely available on the Web sites of PLoS Medicine at http://www.plosmedicine.org/, Annals of Internal Medicine at http://www.annals.org/, and Epidemiology at http://www.epidem.com/). Information on the STROBE Initiative is available at www.strobe-statement.org.

For peer review only

Adult psychosocial outcomes of men and women who were looked-after or adopted as children: prospective observational study

Journal:	*BMJ Open*
Manuscript ID	bmjopen-2017-019095.R1
Article Type:	Research
Date Submitted by the Author:	05-Dec-2017

Complete List of Authors:	Teyhan, Alison; Univ Bristol, Population Health Sciences, Bristol Medical School<br>Wijedasa, Dinithi; Univ Bristol, School for Policy Studies<br>Macleod, John; University of Bristol, Population Health Sciences, Bristol Medical School

**Primary Subject Heading**:	Epidemiology
Secondary Subject Heading:	Mental health, Smoking and tobacco, Addiction
Keywords:	Adoption, Looked-after child, Adult outcomes, Substance use, MENTAL HEALTH, ALSPAC

SCHOLARONE™
Manuscripts

Adult psychosocial outcomes of men and women who were looked-after or adopted as children: prospective observational study

A Teyhan[1*], D Wijedasa[2], J Macleod[1]

[1]Population Health Sciences, Bristol Medical School, University of Bristol, UK

[2]School of Policy Studies, University of Bristol, UK

*Corresponding author: alison.teyhan@bristol.ac.uk

For peer review only

Abstract

Objective

To investigate whether men and women who were looked-after (in public care) or adopted as children are at increased risk of adverse psychological and social outcomes in adulthood.

Design, setting

Prospective observational study using the Avon Longitudinal Study of Parents and Children, which recruited pregnant women and their male partners in and around Bristol, UK in the early 1990s.

Participants

8775 women and 3654 men who completed questionnaires at recruitment (mean age: women 29; men 32) and five years later.

Exposure

Childhood public care status: looked-after; adopted; not looked-after or adopted (reference group).

Outcomes

Substance use (alcohol, cannabis, tobacco) pre-pregnancy and five years later; if ever had addiction; anxiety and depression during pregnancy and five years later; if ever had mental health problem; social support during pregnancy; criminal conviction.

Results

For women, 2.7% were adopted and 1.8% had been looked-after; for men, 2.4% and 1.8% respectively. The looked-after group reported the poorest outcomes overall, but this was not a universal pattern, and there were gender differences. Smoking rates were high for both the looked-after (men 47%, women 58%) and adopted (men 44%, women 40%) groups relative to the reference group (both 28%). The looked-after group were at increased risk of a high depression score (men: 26% versus 11%, OR 2.9 [95% CI 1.5-5.6]; women: 24% versus 9%, 3.4 [2.2-5.0]). A high anxiety score was reported by 10% of the reference women, compared to 26% of those looked-after (3.0 [2.0-4.5]) and 17% of those adopted (1.8 [1.2-2.6]). Looked-after men and women reported the lowest social support, and criminal convictions and addiction were highest for looked-after men. Adjustment for adult socio-economic position generally attenuated associations for the looked-after group.

Conclusions

The needs of those who experience public care as children persist into adulthood. Health and social care providers should recognise this.

For peer review only

Strengths and Limitations

- The longitudinal, population-based cohort allows comparison of several psychosocial outcomes in adults who experienced being looked-after or adopted as children.
- Several of the outcomes are measured at two time-points five years apart, allowing the persistence or amelioration of disadvantage to be considered.
- We have several measures of adult socio-economic position, but do not have data on early life factors.
- The cohort only includes parents, which limits generalisability to the wider population of looked-after and adopted adults.
- Adoption and social care practices in England have changed since our participants were children, mainly in the 1960s and 1970s. Therefore the results may not be generalisable to those who have been adopted or looked-after more recently.

For peer review only

1. Introduction

In the UK, the state has a duty to safeguard and promote the welfare of children, to protect them from maltreatment, and to promote their health and development (1). Surveillance by frontline services aims to identify children in need. Their families can be provided with additional support, but if this is inadequate to mitigate risk or to enable the child's needs to be met, parental responsibility may be taken on by the state. Reasons for this include abuse, neglect, family dysfunction, acute family stress, and disability. Children may be looked-after on a short or long-term basis, with some experiencing multiple periods of care (2). Previous research has estimated that the chances of returning home after a year in care are very small; about eight out of ten of those who have been looked-after for a year are still looked-after one year later (3).

For most children who need a permanent substitute home in the UK, adoption or long-term fostering becomes the plan. For younger children, adoption is often the preferred long-term care model as it provides a greater level of permanence and a 'family for life' (4, 5). Children who grow up with adopted parents generally report higher levels of emotional security, sense of belonging, and well-being than those in long-term foster care (6).

For children in long-term care who age-out of the care system, the transition to adulthood can be fraught with difficulties. In England this happens at age 18, with the local authority continuing to provide support and advice until age 21, or 25 if in education or training (7). Beyond this, these young people often have little financial or social support. Their transition to adulthood is accelerated compared to their peers; they have to live independently, find employment, and manage their finances at a younger age, and most often, without the support of a stable family (8).

Routine statistics and epidemiological studies show that children in care in the UK do worse than their peers across many domains. Notably, they have lower educational attainment (9-12), poorer mental health (13-16), and are over-represented in the criminal justice system (17). They are at increased risk of many inter-linked adverse circumstances as they enter adulthood, including unemployment, homelessness, social isolation, drug use, self-harm, and imprisonment (8, 18, 19). These risks may be reduced if children are successfully adopted, however some studies have found that adopted children have more emotional, behavioural, and academic problems than their non-adopted peers (20), and experience more bullying in adolescence (21).

Relatively little research has considered outcomes beyond young adulthood. Two previous studies have used UK cohort data to examine the impact of being looked-after on outcomes in adulthood: one used the 1970 British Birth Cohort Study (BCS70) to examine outcomes in men and women at age 30 (22), the other used the Millennium Cohort Study (MCS) to examine outcomes in women mostly aged 20-39, who were born in the 1960s and '70s (23). In both, those who had been in care had increased risk of poorer outcomes in adulthood e.g. lower socio-economic status, poorer health, and more smoking. A further British Cohort Study, the 1958 BCS, has been used to compare outcomes in adulthood between those who were adopted and their peers (24, 25), with the adopted women in particular

For peer review only

being found to have positive outcomes. Surprisingly, there have been no UK studies that have compared adult outcomes of children who were adopted out of care versus those in other care placements, despite the fact that both are radical interventions in the lives of children.

Therefore although the poor outcomes of care leavers in the UK are well-recognised, including by the Government (26), little is known about how such adversities persist or change for this vulnerable group beyond young adulthood. Similarly, although many adopted children do well, there has been little research on how their outcomes in adulthood compare to their looked-after or general population peers. As a consequence, there is a lack of evidence on the additional needs, if any, of care leavers and adoptees at older ages. This paper aims to add to this currently limited evidence base. We use data from another UK population-based cohort study whose adult participants were born mostly in the 1960s. This cohort allowed the examination of outcomes at two time-points in adults who were looked-after and adults who were adopted as children. We chose outcomes which relate to four key areas in which looked-after children or young care leavers are known to experience increased risk: mental health difficulties, substance use, poor social support, and criminal conviction.

2. Methods

Sample

The Avon Longitudinal Study of Parents and Children (ALSPAC) recruited 14,541 pregnant women living in a defined area in and around the city of Bristol, UK in 1991-1992. The women have been sent regular postal questionnaires ever since. They were also sent questionnaires for their partner to complete. The women chose who, if anyone, to give these to. For a minority of the women, the partner she gave the questionnaires to changed over time. We therefore only include men in our study who consistently report being the father of the study child to ensure we have data on the same man at all time points. The main results presented in this study are based on 8775 women and 3654 men who returned questionnaires during the pregnancy and when the study child was aged 5 years. More details on ALSPAC are available in the cohort profiles (27, 28), and the searchable data dictionary (www.bristol.ac.uk/alspac/researchers/).

Exposure measures

Experiences of being in care or adopted were reported via questionnaires administered during the pregnancy: adopted when aged <18 years (no, yes); ever been in the care of a local authority or voluntary agency (no; yes; unsure); ever stayed in a children's or residential home (no; <1 week; 1week – 1 month; 1-6 months; >6 months); ever stayed in a foster parents' home (no; yes). Those who responded yes to having been in care, or to having lived in a foster or children's home, were deemed to have been in care. A 3-category exposure variable was derived defining childhood public care status: not looked-after or adopted; looked-after (not adopted); adopted. For the main analyses, the few individuals

For peer review only

who reported that they were unsure if they had ever been looked-after (and who did not report that they had lived in children's home or foster care), were included in the looked-after group. A sensitivity analysis was also conducted excluding these individuals.

Main outcome measures

Substance use

Pre-pregnancy substance use was reported via questionnaires administered during the pregnancy with the study child: smoked regularly (no, yes); drank at least one unit of alcohol per day (no, yes) [1 UK unit is 8 grams of alcohol e.g. half a pint of lager (29)]; used cannabis in the 6 months before pregnancy (no, yes). Participants also reported if they had ever suffered from drug addiction or alcoholism (no, yes). Five years later, participants reported if they: currently smoked; had used cannabis in the past year; currently drank at least one unit of alcohol per day.

Mental health

During pregnancy, the participants reported if they had ever had schizophrenia, anorexia, severe depression, or other psychiatric problem: a variable was derived stating whether the respondent had ever had any of these psychiatric problems (no, yes). Symptoms of depression and anxiety were measured at 18 weeks gestation. Depressive symptoms were measured using the Edinburgh Postnatal Depression Scale. Although this measure was originally designed for use with postnatal women, the 10 items are not specific to women or this period and it has been validated for use at other times (30). Anxiety symptoms were measured by the 8 items of the anxiety subscale of the Crown-Crisp Experiential Index (31). Binary variables were derived which determined whether a respondent had a high score (>90% percentile) or not: for women this represented a score ≥14 for depression and ≥10 for anxiety; for men a score ≥10 for depression and ≥7 for anxiety. The women also reported these measures in the same way when their child was aged 5 years. At this time-point, both women and men reported if they had experienced symptoms of anxiety/nerves or depression in the past year.

Social support and networks

During the pregnancy, the participants reported their social support via their level of agreement to ten statements, and their social network via a further ten statements (details on question wording in supplementary Tables A and B). For both measures, scores were summed with each having a possible range of 0 to 30. A higher score reflects more social support or a better social network. Binary variables were derived to identify those with poor social support or networks, defined as being in the bottom decile. Cut-offs were the same for men and women: ≤12 for low social support, ≤18 for poor social network.

Conviction for criminal offence

Participants were asked if they had ever been convicted of a criminal offence in several questionnaires which covered the period from the start of pregnancy, up until the child was aged 5. A binary variable was derived; any conviction (no, yes).

For peer review only - http://bmjopen.bmj.com/site/about/guidelines.xhtml

Other variables

Socio-economic position (SEP) were measured during the pregnancy. Household occupational social class was based on the lowest class reported by the woman and her partner (I/II [professional/managerial & technical]; III [skilled manual and skilled non-manual]; IV/V [semi-skilled/unskilled manual] (32)). Men and women also reported their highest educational qualification (university degree; A level; O level; vocational/none). Other measures were reported by women only: financial difficulties (quartiles of score with range 0–40, where 0 is no financial difficulties); housing tenure (owned/mortgaged, private rent, council rent, other); partner status (married; live with partner; do not live with partner; no partner); whether pregnancy with the study child was intentional (no, yes); and their parity (0; 1; 2; 3+). In the 5-year questionnaire, they reported their pregnancy intentions (not pregnant; intend to try later; currently trying; pregnant).

The highest childhood happiness of the men and women was derived from responses to questions on how happy their childhood was at 0-5 years, 6-11 years and 12-15 years (very happy; moderately; quite or very unhappy). School stability was measured by the number of schools attended before the age of 16 years (0-2; 3; 4; 5+). Participants also reported adversities before the age of 17 years: suspended from school; in trouble with police, pregnant (or partner pregnant for the men).

Analysis

The exposure groups were compared in terms of their adult SEP and childhood experiences using descriptive statistics. Logistic regression models were used to examine associations between the exposure and each of the substance use, mental health, social network, and criminal conviction outcomes. Those who had not been adopted or looked-after were the reference group. Models were run unadjusted and adjusted for age and measures related to SEP (relationship status, education, financial difficulties, social class and housing tenure). Models also adjusted for parity for women, and for whether the pregnancy was intentional for the pregnancy time-point outcomes, and for pregnancy intentions for the 5-year time-point outcomes. Where results are described as being 'adjusted' in the results text, this means after adjustment for age, all of the SEP variables, plus parity and pregnancy-intention variables where relevant. All analyses were performed stratified by gender.

A sensitivity, cross-sectional analysis of the measures reported during pregnancy only was performed to determine if results were consistent when the sample was not restricted to those who also participated 5 years later.

Missing data

Of the 8775 women in the longitudinal sample, 46.7% had complete data and a further 43.8% had between 1 and 4 missing values. Of the 3654 men, 16.5% had complete data, and a further 78.2% had 1-4 missing values. The percentage of missing data is summarised for each variable in supplementary Table C. Multiple imputation using chained equations was used to replace missing data with predictions based on information observed in the sample.

For peer review only

All of the variables included in the analyses models, plus other variables associated with missingness or the variables in the model, were included in the imputation models. Imputed datasets (55 for the women, 100 for the men) were created and analysed using mi estimate commands in Stata 14.2 (StataCorp, College Station, Texas).

In the cross-sectional sample for the sensitivity analysis, complete-case analysis was performed. Of the 11,571 women who reported their childhood care status, 7795 had complete outcome data reported during pregnancy, and of those 7088 had complete confounder data. Of the 7676 men who reported childhood care status, 3163 had complete outcome data reported during their partner's pregnancy, of whom 2820 had complete confounder data.

3. Results

In the longitudinal sample, 2.4% (95% CI 1.9% to 2.9%) of the men and 2.7% (2.4% to 3.1%) of the women had been adopted. A further 1.4% (1.0% to 1.8%) of the men and 1.8% (1.5% to 2.1%) of the women had been looked-after. These looked-after percentages include the small number of participants who reported that they were unsure if they had been looked-after; excluding them reduced the proportion who were looked-after to 1.1% of men and 1.6% of women.

Of the men who reported their care setting whilst being looked-after, 39% had lived in foster care only, 44% in a children's home only, and 17% in both. Of the women, 30% had lived in foster care, 42% in a children's home, and 28% in both. Over 60% of the men and women who had lived in a children's home had done so for >6 months. Of the adoptees, many had been adopted aged <1 year (women 49%, men 63%). A minority of the adoptees had also been in care (women 14%, men 16%), with a similar proportion reporting that they didn't know.

Compared to the reference group, those who had been looked-after were generally younger, less likely to be married, and of lower SEP (women Table 1; men Table 2). With regards their childhood, they were the least likely to have been very happy, and the most likely to have attended multiple schools, to have been suspended, and in trouble with the police (supplementary Table D). Women who had been looked-after were the most likely to have been pregnant before the age of 17, and to have had several previous births. They were least likely to report the pregnancy with the study child was intentional, but most likely to be trying to conceive 5 years later. Participants who had been adopted generally had SEP and childhood measures intermediate to those who had been looked-after and those who had not been looked-after or adopted.

Substance Use (Table 3)

Those who had been looked-after or adopted were more likely to be smokers than the reference group at both time-points, with the highest rates observed for looked-after women. Women who had been looked-after or adopted were also more likely to have used cannabis at both time-points. Daily alcohol consumption did not differ between those who

For peer review only

had been looked-after and those in the reference group for men or women. Adopted women were the most likely of the women to drink daily pre-pregnancy, but the adopted men were the least likely at both time-points. Rates of addiction to alcohol or drugs were particularly high in men who had been looked-after or adopted. In women, addiction was also more common in those who had been looked-after or adopted, but numbers were small. Adjustment for age, relationship status, and measures of SEP attenuated associations observed for smoking for participants who were looked-after, but had less of an effect on alcohol and cannabis associations, or associations for participants who were adopted.

Mental Health (Table 4)

Women who had been looked-after were the most likely to have high depression and anxiety scores at both time-points, to have ever had a mental health problem, and to have experienced symptoms of anxiety and depression in the past year at the later time-point. Women who had been adopted were also more likely to have high anxiety scores, and to have had a mental health problem, than those in the reference group. Adjustment attenuated results for the looked-after group but not the adopted group. Men who had been looked-after were more likely to have a high depression score during their partner's pregnancy, and more likely to have ever had a mental health problem, than the reference group. Adjustment for SEP attenuated the differences. At the five-year time-point, there was no difference by care status in the percentage of men reporting symptoms of anxiety or depression in the previous year. Men in each category were less likely to report these symptoms than women, with the gender difference being particularly large for depressive symptoms.

Social support and conviction for criminal offence (Table 5)

Participants who had been looked-after were the most likely to report low social support and a limited social network. Differences relative to the reference group were attenuated on adjustment. Women who had been adopted were more likely to report a poor social network than the reference group, but not a low level of social support. Adopted men did not differ from the reference men.

Men and women who had been looked-after or adopted were more likely to have been convicted of an offence compared to the reference groups. Differences were attenuated on adjustment. Conviction rates were considerably higher for men than women in each of the exposure categories.

Sensitivity analyses

Analyses were repeated excluding the small number of participants who were unsure if they had been looked-after and results were consistent with those of the main analyses. In cross-sectional, complete-case analyses of the outcomes reported in pregnancy only, a slightly higher proportion of the men had been looked-after (2.4%) or adopted (3.2%) compared to the longitudinal sample. In contrast, proportions for women in the cross-sectional sample (looked-after 1.6%; adopted 2.6%) were similar to those in the longitudinal sample. The men in the complete case sample were of lower SEP than those in the

For peer review only

longitudinal sample (supplementary Table E), but this was not observed for the women (supplementary Table F). Overall, the pattern of results we observed in the longitudinal sample was broadly replicated (supplementary Tables G to I).

Discussion

Overview of findings

We used a population-based study to examine substance use, mental health, social support, and criminal conviction in adulthood amongst individuals who had been looked-after or adopted as children, compared with their peers in the sample who were neither adopted or looked-after. There was a general, but not universal, pattern of these adults reporting more mental health problems, smoking, and criminal convictions, and less social support, than their peers. Overall, the looked-after individuals reported more negative outcomes than those who had been adopted. There were gender differences in some of the associations observed. For example, women who had been looked after had high rates of anxiety, whereas men who had been looked-after had an excess risk of addiction and criminality. Adjustment for SEP measures often attenuated associations substantially for the looked-after group, but generally had less of an impact for the adopted group. Rather than implying that adult SEP confounds the relationship between childhood care status and adult psychosocial outcomes, this suggests that it in part mediates these effects.

Comparison to previous literature – adults who have been looked-after

In the UK, the poor outcomes for young care leavers have been widely reported, both in academic literature and increasingly in mainstream media (e.g. 33, 34-37). Our study is one of the few to consider outcomes at an older age. The high rate of depressive symptoms in adults who had been looked after as children in our sample was in concordance with both the BCS70 (22) and MCS (23) studies. Neither included measures of anxiety with which to corroborate our finding of high anxiety in looked-after women but not men.

Whilst the looked-after men reported more addiction in our sample, it was for the looked-after women that we found the strongest association with cannabis use. In a qualitative study of care leavers in England, cannabis was viewed as ‘relatively harmless’, ‘acceptable’, and ‘to have little impact on parenting’: some women said it helped them cope with the stress of looking after a baby (8). This could explain our finding of a similar prevalence of cannabis use pre and post pregnancy, in contrast to the decline observed for smoking. Less than 10% of the looked-after individuals in ALSPAC reported cannabis use, a considerably lower percentage than observed in the young care leavers, and in agreement with their general finding that substance use declines with age. The numbers reporting use of other illegal substances was too small to analyse, however the ‘ever had an addiction’ measure is likely to include addictions to other illegal substances. The BCS70 study included a measure of ‘illegal substance use in past year’, without further breakdown, and found high levels in the men compared to ALSPAC; 26% of those never in care and 34% of those who had been in care (22). Part of the discrepancy in drug use prevalence between the two studies could

For peer review only

reflect differences in sample selection: the ALSPAC sample were recruited to the study as adults who were expecting a baby, whereas the BCS70 sample were in that study from birth, as it was their parents who were recruited.

Smoking rates were very high for looked-after men and women in ALSPAC, consistent with previous studies: of the looked-after women in the MCS, 73% had ever been smokers and 58% smoked during pregnancy (23); in the care leavers study, two thirds were daily smokers (8). In contrast, we did not find daily alcohol consumption to be associated with looked-after status in ALSPAC. Our measure does not capture binge drinking behaviours, and reverse causation could be an issue whereby those with alcohol problems had stopped drinking. However our results are consistent with the BCS70 study, where adults who had been looked-after were no more likely to have problems with alcohol (22).

The looked-after men and women in ALSPAC were the most likely to report a poor social network and limited social support. The instability of life in care can make it difficult to build and maintain friendships and a support network, and young people can reach adulthood with no family, and no social base (6). This reduced social capital exacerbates their difficulties in transitioning to a successful, independent adult life. Our results show that reduced social capital can persist for many years.

The majority of looked-after children in the UK do not receive a criminal conviction, but as a group they are over-represented in the criminal justice system (17). In our sample, almost a quarter of the looked-after men had been convicted of an offence in the five year period considered. Rates for looked-after women, although lower than the men, were also elevated. Our results highlight that for men in particular, criminal involvement continues for a substantial minority of those who were looked-after, even once they have started a family of their own. Findings were consistent in the BCS70 (22).

Comparison to previous literature – adults who have been adopted

Comparing outcomes in those adopted versus those who remain in the care system, and identifying causal processes, is complicated because those who successfully complete the adoption process may be systematically different from those who remain in long-term care, and these differences may influence their outcomes in addition to any independent influence of the model of care they receive. The adult SEP of the adoptees in ALSPAC was higher than the looked-after group but lower than the reference group. In contrast, adopted women in the 1958 British birth cohort had a higher SEP than the general population at age 33, but this was not true for the men (24).

For substance use, no clear pattern emerged when comparing those who had been adopted to those who had been in care. Smoking prevalence and cannabis use were similar for the men, and addiction was similar for the women. Of all the groups, daily alcohol consumption was consistently lowest in the adopted men, whereas of the women those who had been adopted were the most frequent drinkers pre-pregnancy. For men and women in the British 1958 cohort, alcohol problems did not differ between adoptees and the general population (24). Another study used the 1958 cohort data, and similarly found no difference in alcohol abstinence, but the adoptees were more likely to have smoked than the general population.

For peer review only

This study also included an additional comparison group, adults who had been adopted from Hong Kong orphanages by British parents; this group were the least likely to drink alcohol or smoke (25).

Adoptees in ALSPAC were more likely to report having ever had a mental health problem than the reference group, but less likely than the looked-after group; in contrast the adopted men and women in the 1958 birth cohort did not report excess past emotional problems (24). Adopted women in ALSPAC had high anxiety scores at both time-points relative to the reference group, but again this was not as high as that of the looked-after women. The adopted men did not have higher anxiety scores than the reference men, and neither gender had excess risk of depression. In the 1958 cohort the adoptees did not have a higher risk of current depressive symptoms (24) or other mental health problems (25).

Adopted men and women in our study were not more likely than the reference group to have low social support, but the adopted women were more likely to have a limited social network albeit not to the extent observed for the looked-after women. In the 1958 British cohort there was a gender difference: adopted women had the highest level of social support, but adopted men the lowest (24).

The preference for adoption versus long term foster care differs between countries (38, 39), and there are many other differences internationally in child protection systems, and cultural and social norms regarding out-of-home care and adoption. An added complication when considering adult outcomes is that the exposure happened many years before, and care and adoption systems have changed over time within and between countries. For these reasons, we have focused our paper on the UK context. However adult outcomes of those who have been in care or adopted have been studied in other countries, including Sweden (40-42) and the US (43).

Role of early childhood adversity

Many looked-after children have experienced adverse childhood experiences (ACEs), which are associated with poor long term outcomes irrespective of whether the children experiencing these adversities are subsequently looked-after (44). For many children being looked-after or adopted is likely to be beneficial. For example, educational attainment may improve after long-term care (10). The generally worse outcomes we have found in adults who have been in care should not be interpreted as strong evidence of adverse effects of care itself. Rather, having been in care is a marker of substantial early childhood disadvantage. A recent paper which examined ACEs in the ALSPAC women, without considering childhood care status, found higher levels of mental illness and smoking, and poorer education and social support, in those who had experienced maltreatment in childhood (45). The results mirror ours, and provide evidence for ACEs being important factors in the associations we observe. We were unable to directly examine the role of ACEs in our study as it is not possible to determine if the measures relate to a time before or after a child entered the care system. It is important to acknowledge that becoming looked-after is not inevitably positive for children. Some aspects of care, for example experiences of

For peer review only

abuse or neglect within the care system, may compound the effects of early childhood adversity (46).

Strengths and Limitations

Strengths of our study include its basis in the general population and our ability to examine outcomes at two points in adulthood in participants who reported being looked after compared to those who reported being adopted. Including outcomes at two points in time allowed examination of stability and change in the outcomes we considered. Our study also has limitations that we acknowledge. ALSPAC does not include adults who are not parents. To be eligible, women needed to be pregnant, and men needed to be invited by their pregnant partner to participate. Adults with more problematic lives may be underrepresented in our sample. In women this could reflect lower engagement with antenatal services, through which ALSPAC recruited its sample. In men it is possible that those who had a difficult or unstable relationship with their child's mother will be less likely to be in the study. These considerations lead us to expect that the associations we found will be under-estimates of those in the wider looked-after community. The participants were children mostly in the 1960s and 70s, and care procedures have changed over time, notably in the greater use of foster as opposed to group care (4), and extended support until age 25 (47). Furthermore, whereas in the past many adoptions were of relinquished babies, now most are of children aged 1-4 years who are in care due to neglect or abuse (48). Therefore our results may not be generalisable to those currently in care, or adopted in the last three decades. The ALSPAC measures on looked-after status and adoption lack detail. The age a child enters care, and the number of placements they have, are thought to be key factors in determining the likelihood of positive outcomes (49), but we do not have this information on our participants. We do not know the reason for their care status, or the age they left the care system. Some of the adoptions may have been by step-parents, rather than as a result of being removed from birth parents. Some of the adopted individuals had also been in care, but we do not know why or for how long. As only a small percentage of children experience care or adoption, numbers in a population-based cohort, even one as large as ALSPAC, will be small.

Implications for practice and policy

Our results are likely of most use to health and social care providers, and evidence a need for support mechanisms to be in place for care leavers beyond young adulthood. Our data do not allow us to made recommendations beyond this. As discussed, we have limited information on the type of care received, at what age, or how long for. We therefore cannot make policy recommendations with regards to these factors. But what we can conclude is that those who experience the care system continue to have poorer outcomes in adulthood than their peers, many years after they have left the care system, and to when they have children of their own. We have also shown that adopted individuals have excess risk in some areas, including smoking, addiction, and mental health.

Long-term, continued improvements to the care system are needed to maximise the life chances of future children who experience adversity in childhood and are not able to grow

For peer review only

up with their birth parents. In particular, evidence is needed on which modifiable aspects of care, beyond permanence and placement stability, promote better outcomes. In the meantime, it must be recognised that some of today's adults who experienced the care system as children have higher needs than those in the general population. In particular, our results suggest a particular need for mental health care, social and educational support, and for services to reduce the harms associated with substance use.

By highlighting the currently limited evidence base in this area, and the limitations of the ALSPAC data, we hope that our work will also encourage other researchers with suitable data to consider undertaking similar analyses. A stronger evidence base would ultimately allow more specific policy recommendations to be made with the ultimate goal of improving the long-term life chances of those unable to grow up with their birth families.

For peer review only

References

1. HM Government. Working together to safeguard children. A guide to inter-agency working to safeguard and promote the welfare of children. 2015 [Available from: www.gov.uk/government/uploads/system/uploads/attachment_data/file/592101/Working_Together_to_Safeguard_Children_20170213.pdf.
2. Mc Grath-Lone L, Harron K, Dearden L, Nasim B, Gilbert R. Data Resource Profile: Children Looked After Return (CLA). International Journal of Epidemiology. 2016;45(3):716-7f.
3. Sinclair I, Baker C, Lee J, Gibbs I. The Pursuit of Permanence. London: Jessica Kingsley Publishers; 2007.
4. Department for Education, National Statistics. Children looked after in England (including adoption) year ending 31 March 2016 (SFR 41/2016).2016; (Accessed May 2017). Available from: https://www.gov.uk/government/uploads/system/uploads/attachment_data/file/556331/SFR41_2016_Text.pdf.
5. Department for Education. Adoption: A Vision for Change 2016 [Available from: https://www.gov.uk/government/uploads/system/uploads/attachment_data/file/512826/Adoption_Policy_Paper_30_March_2016.pdf.
6. Triseliotis J. Long-term foster care or adoption? The evidence examined. Child & Family Social Work. 2002;7(1):23-33.
7. GOV.UK. Leaving foster or local authority care [Available from: www.gov.uk/leaving-foster-or-local-authority-care.
8. Ward J, Henderson Z, Pearson G. One problem among many: drug use among care leavers in transition to independent living. 2003.
9. Department for Education, National Statistics. Outcomes for Children Looked After by Local Authorities in England, as at 31 March 2016 (SRF 12/2017). 2017 [Available from: https://www.gov.uk/government/uploads/system/uploads/attachment_data/file/602087/SFR12_2017_Text.pdf.
10. Fletcher J, Strand S, Thomas S. The Educational Progress of Looked After Children in England.

Technical Report 1: Secondary School Progress and Attainment 2015 [Available from: http://reescentre.education.ox.ac.uk/wordpress/wp-content/uploads/2015/11/EducationalProgressLookedAfterChildrenTechnical-Report-1_Nov2015.pdf.
11. The Scottish Government. Education Outcomes for Looked After Children 2014/15 2015 [Available from: http://www.gov.scot/Resource/0050/00501939.pdf.
12. O'Higgins A, Sebba J, Luke N. What is the relationship between being in care and the educational outcomes of children? An international systematic review 2015 [Available from: http://reescentre.education.ox.ac.uk/wordpress/wp-content/uploads/2015/09/ReesCentreReview_EducationalOutcomes.pdf.
13. FORD T, VOSTANIS P, MELTZER H, GOODMAN R. Psychiatric disorder among British children looked after by local authorities: comparison with children living in private households. The British Journal of Psychiatry. 2007;190(4):319-25.
14. Memarzia J, St Clair MC, Owens M, Goodyer IM, Dunn VJ. Adolescents leaving mental health or social care services: predictors of mental health and psychosocial outcomes one year later. BMC Health Services Research. 2015;15(1):185.
15. Beagley E, Hann G, Al-Bustani N. G156(P) Mental Health Needs of Looked After Children in One of the Most Deprived Boroughs in England. Archives of Disease in Childhood. 2014;99(Suppl 1):A69-A.
16. Minnis H, Everett K, Pelosi AJ, Dunn J, Knapp M. Children in foster care: Mental health, service use and costs. European Child & Adolescent Psychiatry. 2006;15(2):63-70.

For peer review only

17. Prison Reform Trust. In Care, Out of Trouble 2016. Available from: http://www.prisonreformtrust.org.uk/Portals/0/Documents/In%20care%20out%20of%20trouble%20summary.pdf.
18. Schofield G, Biggart L, Ward E, Larsson B. Looked after children and offending: An exploration of risk, resilience and the role of social cognition. Children and Youth Services Review. 2015;51:125-33.
19. Silver M, Golding K, Roberts C. Paper 9: Delivering psychosocial services for children, young people and families with complex social care needs. 2015 [cited Accessed May 2017]. In: The Child and Family Clinical Psychology Review [Internet]. The British Psychological Society, [cited Accessed May 2017]. Available from: https://shop.bps.org.uk/publications/child-and-family-clinical-psychology-review-no-3-summer-2015.html.
20. Brodzinsky DM. Long-Term Outcomes in Adoption. The Future of Children. 1993;3(1):153-66.
21. Wijedasa D, Selwyn J. Transition to adulthood for young people in adoptive care: Secondary analysis of data from the longitudinal study of young people in England (LYSPE). Hadley Centre for Adoption and Foster Care Studies; 2011 [Available from: www.bristol.ac.uk/media-library/sites/sps/migrated/documents/transitiontoadulthood.pdf.
22. Viner RM, Taylor B. Adult Health and Social Outcomes of Children Who Have Been in Public Care: Population-Based Study. Pediatrics. 2005;115(4):894-9.
23. Botchway SK, Quigley MA, Gray R. Pregnancy-associated outcomes in women who spent some of their childhood looked after by local authorities: findings from the UK Millennium Cohort Study. BMJ Open. 2014;4(12).
24. Collishaw S, Maughan B, Pickles A. Infant adoption: psychosocial outcomes in adulthood. Social Psychiatry and Psychiatric Epidemiology. 1998;33(2):57-65.
25. Rushton A, Grant M, Feast J, Simmonds J. The British Chinese Adoption Study: orphanage care, adoption and mid-life outcomes. Journal of Child Psychology and Psychiatry. 2013;54(11):1215-22.
26. Cameron D. Prime Minister: Conference speech 2015 2015 [Available from: http://press.conservatives.com/post/130746609060/prime-minister-conference-speech-2015.
27. Fraser A, Macdonald-Wallis C, Tilling K, Boyd A, Golding J, Smith GD, et al. Cohort profile: the Avon Longitudinal Study of Parents and Children: ALSPAC mothers cohort. International journal of epidemiology. 2013;42(1):97-110.
28. Boyd A, Golding J, Macleod J, Lawlor DA, Fraser A, Henderson J, et al. Cohort profile: the 'children of the 90s'—the index offspring of the Avon Longitudinal Study of Parents and Children. International journal of epidemiology. 2012:dys064.
29. NHS Choices. Alcohol Units 2015 [Available from: http://www.nhs.uk/Livewell/alcohol/Pages/alcohol-units.aspx.
30. Thorpe K. A study of the use of the Edinburgh Postnatal Depression Scale with parent groups outside the postpartum period. Journal of Reproductive and Infant Psychology. 1993;11(2):119-25.
31. Birtchnell J, Evans C, Kennard J. The total score of the Crown-Crisp Experiential Index: A useful and valid measure of psychoneurotic pathology. British Journal of Medical Psychology. 1988;61(3):255-66.
32. Office for Population Censuses & Surveys. Standard Occupational Classification. London; 1991.
33. John-Baptiste A. Leaving care: The financial reality: BBC News; 2017 [Available from: http://www.bbc.co.uk/news/uk-39455547.
34. Greenwood G. Early deaths among care leavers revealed BBC News2017 [Available from: http://www.bbc.co.uk/news/uk-38961818.
35. Slawson N. Girls in state care and care leavers still at high risk of teenage pregnancy. The Guardian. 2016.
36. BBC. Kicked Out: From Care to Chaos 2017 [Available from: http://www.bbc.co.uk/programmes/p04xwl4l.

For peer review only

37. BBC Radio 4. Abandoned to their fate [Internet]; 2015 7 June 2015. Podcast. Available from: http://www.bbc.co.uk/programmes/b05wy64q
38. Parton N. International comparison of child protection systems: University of Huddersfield; 2010 [Available from: http://eprints.hud.ac.uk/id/eprint/12187/.
39. Munro ER, Manful E. Safeguarding children: a comparison of England's data with that of Australia, Norway and the United States 2012 [Available from: www.gov.uk/government/uploads/system/uploads/attachment_data/file/183946/DFE-RR198.pdf.
40. Berlin M, Vinnerljung B, Hjern A. School performance in primary school and psychosocial problems in young adulthood among care leavers from long term foster care. Children and Youth Services Review. 2011;33(12):2489-97.
41. Vinnerljung B, Hjern A. Cognitive, educational and self-support outcomes of long-term foster care versus adoption. A Swedish national cohort study. Children and Youth Services Review. 2011;33(10):1902-10.
42. Smyer MA, Gatz M, Simi NL, Pedersen NL. Childhood adoption: Long-term effects in adulthood. Psychiatry. 1998;61(3):191-205.
43. Kessler RC, Pecora PJ, Williams J, et al. Effects of enhanced foster care on the long-term physical and mental health of foster care alumni. Archives of General Psychiatry. 2008;65(6):625-33.
44. Bellis MA, Lowey H, Leckenby N, Hughes K, Harrison D. Adverse childhood experiences: retrospective study to determine their impact on adult health behaviours and health outcomes in a UK population. Journal of Public Health. 2014;36(1):81-91.
45. Plant DT, Jones FW, Pariante CM, Pawlby S. Association between maternal childhood trauma and offspring childhood psychopathology: mediation analysis from the ALSPAC cohort. The British Journal of Psychiatry. 2017.
46. Biehal N, Cusworth L, Wade J, Clarke S. Keeping children safe: allegations concerning the abuse or neglect of children in care. University of York: NSPCC; 2014 [Available from: www.nspcc.org.uk/globalassets/documents/evaluation-of-services/keeping-children-safe-report.pdf.
47. UK Government. Children (Leaving Care) Act 2000 2000 [Available from: http://www.legislation.gov.uk/ukpga/2000/35/contents.
48. Adoption UK. Adoption facts and figures for England 2017 [Available from: www.adoptionuk.org/press-media/adoption-facts-and-figures-england.
49. Jones R, Everson-Hock ES, Papaioannou D, Guillaume L, Goyder E, Chilcott J, et al. Factors associated with outcomes for looked-after children and young people: a correlates review of the literature. Child: Care, Health and Development. 2011;37(5):613-22.

For peer review only

Footnotes

Contributors: AT conceived the study, conducted the analyses, interpreted the data, and drafted the manuscript. JM helped develop the research question, interpreted the data, and critically revised the paper. DW interpreted the data and critically revised the paper. All authors have read and approved this final version. AT will serve as guarantor for the contents of this paper.

Competing interests JM is a foster carer. AT and DW have no competing interests.

Funding The UK Medical Research Council and the Wellcome Trust (Grant Ref: 092731) and the University of Bristol provide core support for ALSPAC. AT and JM are supported by PEARL (Project to Enhance ALSPAC through Record Linkage), a programme of research funded by the Wellcome Trust (WT086118/Z/08/Z), and The Farr Institute CIPHER, which is supported by a 10-funder consortium: Arthritis Research UK, the British Heart Foundation, Cancer Research UK, the Economic and Social Research Council, the Engineering and Physical Sciences Research Council, the Medical Research Council, the National Institute of Health Research, the National Institute for Social Care and Health Research (Welsh Assembly Government), the Chief Scientist Office (Scottish Government Health Directorates), and the Wellcome Trust (MRC Grant No: MR/K006525/1).

Acknowledgements We are grateful to all the families who took part in ALSPAC, the midwives for their help in recruiting them, and the ALSPAC team, which includes interviewers, computer and laboratory scientists, clerical workers, research scientists, volunteers, managers, receptionists, and nurses.

Ethics approval Ethical approval for ALSPAC was obtained from the ALSPAC Ethics and Law Committee and the Local Research Ethics Committees (LREC). Full LREC details are available online (http://www.bristol.ac.uk/alspac/researchers/research-ethics/). This study was approved by the ALSPAC Executive Committee. It involves secondary analysis of ALSPAC questionnaire data, which is fully anonymised before researchers can access or analyse it.

Data sharing statement The ALSPAC data management plan (available here: http://www.bristol.ac.uk/alspac/researchers/data-access/ documents/alspac-data-management-plan.pdf) describes in detail the policy regarding data sharing, which is through a system of managed open access. Requests for the data used in this paper can be made to the Executive (alspac-exec@bristol.ac.uk).

For peer review only

Table 1: Characteristics of the women by care status in childhood, n=8775

		Reference group (95.5%)	Looked After (1.8%)	Adopted (2.7%)
Age at delivery	Mean age in years	28.8 (28.7-28.9)	27.6 (26.6-28.5)	27.7 (27.2-28.3)
	<=23 years (%)	11.9 (11.2-12.6)	26.7 (19.4-34.1)	15.2 (10.3-20.2)
	>=34 years (%)	15.4 (14.6-16.2)	15.4 (9.3-21.4)	8.7 (5.0-12.5)
Relationship status	Married (%)	80.3 (79.4-81.2)	59.8 (51.4-68.2)	78.3 (72.8-83.8)
	Resident partner (%)	13.4 (12.7-14.2)	20.4 (13.3-27.5)	12.6 (8.1-17.1)
	Non-resident partner (%)	4.4 (4.0-4.9)	14.0 (8.0-20.0)	7.0 (3.5-10.5)
	No partner (%)	1.9 (1.6-2.2)	5.8 (1.7-9.9)	2.1 (0-4.1)
Parity	0 (%)	46.0 (45.0-47.1)	36.3 (28.0-44.7)	48.3 (41.7-54.9)
	3+ (%)	4.6 (4.1-5.1)	15.5 (9.3-21.6)	5.3 (2.4-8.2)
This pregnancy intentional	Yes (%)	73.3 (72.3-74.3)	53.5 (45.0-61.9)	69.2 (63.1-75.4)
Pregnancy intentions at 5 years	Pregnant (%)	3.8 (3.4-4.2)	3.3 (2.5-11.3)	4.8 (1.9-7.8)
	Trying to get pregnant (%)	2.2 (1.9-2.5)	6.9 (2.5-11.3)	1.9 (0-3.7)
Highest maternal education	Degree (%)	15.2 (14.4-16.0)	2.7 (0-5.5)	11.2 (7.0-15.4)
	Vocational/none (%)	24.3 (23.3-25.2)	48.8 (39.8-57.8)	21.8 (16.1-27.4)
Financial difficulties	Q1 (none) (%)	39.4 (38.3-40.4)	22.8 (15.7-29.8)	29.8 (23.6-35.9)
	Q4 (high) (%)	17.7 (16.8-18.5)	33.2 (24.8-41.6)	20.1 (14.7-25.5)
Housing tenure	Owned/mortgaged (%)	80.6 (79.8-81.5)	48.1 (39.6-56.6)	74.8 (69.0-80.6)
Lowest social class of self and partner	I&II (%)	27.9 (26.9-28.9)	16.7 (10.1-23.3)	25.5 (19.6-31.4)
	IV&V (%)	18.1 (17.2-18.9)	26.1 (17.5-34.7)	21.3 (15.6-27.1)

For peer review only

Table 2: Characteristics of the men by care status in childhood, n=3654

		Reference Group (96.1%)	Looked After (1.4%)	Adopted (2.4%)
Age when partner at 18wks gestation	Mean (years)	31.5 (31.3-31.7)	30.5 (29.1-32.0)	30.4 (29.1-31.8)
	<=23 years (%)	3.4 (2.8-4.1)	4.7 (0-11.2)	7.7 (1.7-13.6)
	>=34 years (%)	30.2 (28.6-31.7)	26.8 (13.8-39.9)	23.5 (14.3-32.7)
Relationship status	Married (%)	88.1 (87.0-89.2)	78.8 (67.4-90.3)	78.5 (69.7-87.2)
	Resident partner (%)	10.3 (9.3-11.3)	21.2 (9.7-32.6)	20.4 (11.8-28.9)
	Non-resident partner (%)	1.6 (1.2-2.0)	0	1.2 (0-3.4)
Highest education	Degree (%)	29.3 (27.7-30.8)	4.5 (0-10.8)	18.6 (10.2-27.0)
	Vocational/none (%)	19.3 (17.9-20.6)	53.3 (38.8-67.8)	18.3 (9.9-26.7)
Financial difficulties	Q1 (none) (%)	47.1 (45.5-48.8)	21.9 (10.1-33.7)	33.5 (23.2-43.7)
	Q4 (high) (%)	12.5 (11.4-13.7)	29.2 (16.3-42.1)	19.3 (10.5-28.1)
Housing tenure	Owned/mortgaged (%)	87.4 (86.3-88.5)	53.8 (39.8-67.9)	75.0 (65.8-84.3)
Lowest social class of self and partner	I&II (%)	35.8 (34.2-37.4)	13.8 (4.0-23.7)	21.7 (12.6-30.7)
	IV&V (%)	14.9 (13.7-16.1)	24.9 (12.1-37.7)	17.8 (9.1-26.6)

For peer review only

Table 3: Substance use outcomes by childhood public care status for men and women pre-pregnancy and when study child aged 5 years

		Men			Women		
		%[3]	OR (95% CI)		%	OR (95% CI)	
			Unadjusted	Adjusted[1]		Unadjusted	Adjusted[2]
Has ever had addiction	Reference	2.0	Ref	Ref	1.0	Ref	Ref
	Looked After	13.5	7.8 (3.4-17.8)*	4.0 (1.6-10.0)*	3.1	2.9 (0.9-9.1)	1.6 (0.5-5.4)
	Adopted	6.8	3.6 (1.5-8.6)*	2.3 (0.9-6.0)	3.0	2.9 (1.3-6.8)*	2.8 (1.2-6.6)*
**Pre-pregnancy**							
Smoked regularly	Reference	28.0	Ref	Ref	28.3	Ref	Ref
	Looked After	46.5	2.2 (1.3-3.9)*	1.2 (0.7-2.2)	57.9	3.5 (2.5-4.9)*	1.9 (1.3-2.8)*
	Adopted	44.3	2.0 (1.3-3.2)*	1.7 (1.1-2.7)*	40.3	1.7 (1.3-2.3)*	1.6 (1.2-2.2)*
Drank alcohol daily	Reference	23.8	Ref	Ref	11.3	Ref	Ref
	Looked After	21.8	0.9 (0.5-1.7)	1.1 (0.6-2.3)	9.6	0.8 (0.4-1.5)	0.9 (0.5-1.7)
	Adopted	11.4	0.4 (0.2-0.8)*	0.4 (0.2-0.8)*	17.0	1.6 (1.1-2.3)*	1.8 (1.3-2.6)*
Used cannabis	Reference	6.6	Ref	Ref	3.9	Ref	Ref
	Looked After	9.1	1.3 (0.4-4.7)	1.0 (0.3-3.6)	11.2	3.1 (1.8-5.5)*	2.3 (1.2-4.3)*
	Adopted	9.9	1.5 (0.7-3.4)	1.1 (0.5-2.7)	5.5	1.4 (0.8-2.6)	1.2 (0.6-2.2)
**When study child 5 years**							
Smoked regularly	Reference	22.3	Ref	Ref	23.1	Ref	Ref
	Looked After	44.2	2.8 (1.6-4.8)*	1.6 (0.9-2.9)	52.9	3.7 (2.7-5.3)*	2.0 (1.4-3.0)*
	Adopted	40.3	2.4 (1.5-3.6)*	2.0 (1.2-3.2)*	32.5	1.6 (1.2-2.1)*	1.5 (1.1-2.1)*
Drank alcohol daily	Reference	35.9	Ref	Ref	16.4	Ref	Ref
	Looked After	26.9	0.7 (0.4-1.3)	1.1 (0.6-2.0)	11.0	0.6 (0.4-1.1)	0.9 (0.5-1.6)
	Adopted	19.4	0.4 (0.3-0.8)*	0.5 (0.3-0.9)*	17.0	1.0 (0.7-1.5)	1.2 (0.8-1.7)
Used cannabis	Reference	6.1	Ref	Ref	4.2	Ref	Ref
	Looked After	8.5	1.4 (0.5-3.9)	1.0 (0.3-2.9)	8.8	2.2 (1.2-4.1)*	1.5 (0.7-2.9)
	Adopted	9.9	1.7 (0.8-3.5)	1.2 (0.6-2.6)	8.5	2.1 (1.3-3.5)*	1.9 (1.1-3.1)*

For peer review only

[1]Adjusted for age, relationship status, education, financial difficulties, social class, housing tenure.
[2]Adjusted for age, relationship status, education, financial difficulties, social class, housing tenure, parity, pregnancy intentional (pre-pregnancy models only), pregnancy status/intentions (5 year models only).
[3]Percentages rather than n given as these are results from imputed data, and so the n differs across the imputed datasets. The percentages shown are from results aggregated across all the imputed datasets using Rubin's rules.
*$p<0.05$

For peer review only

Table 4: Mental health outcomes by childhood public care status for men and women during pregnancy and when study child aged 5 years

		Men			Women		
		%[3]	**OR (95% CI)**		%	**OR (95% CI)**	
			Unadjusted	Adjusted[1]		Unadjusted	Adjusted[2]
Has ever had mental health problem	Reference	5.7	Ref	Ref	10.2	Ref	Ref
	Looked After	24.1	5.3 (2.7-10.2)*	3.9 (1.9-7.8)*	25.9	3.1 (2.1-4.5)*	2.2 (1.4-3.2)*
	Adopted	12.4	2.4 (1.2-4.5)*	2.0 (1.0-3.9)	15.5	1.6 (1.1-2.3)*	1.6 (1.1-2.3)*
**During pregnancy**							
High anxiety score (>6m, >9w)	Reference	9.3	Ref	Ref	10.4	Ref	Ref
	Looked After	15.2	1.7 (0.8-3.9)	1.6 (0.7-3.7)	26.1	3.0 (2.0-4.5)*	2.0 (1.3-3.1)*
	Adopted	11.1	1.2 (0.6-2.4)	1.2 (0.6-2.4)	17.4	1.8 (1.2-2.6)*	1.6 (1.1-2.4)*
High depression score (>8m, >13w)	Reference	11.0	Ref	Ref	8.7	Ref	Ref
	Looked After	26.2	2.9 (1.5-5.6)*	2.3 (1.1-4.5)*	24.3	3.4 (2.2-5.0)*	2.0 (1.3-3.1)*
	Adopted	12.3	1.1 (0.6-2.2)	1.0 (0.5-1.9)	10.9	1.3 (0.8-2.0)	1.1 (0.7-1.8)
**When study child 5 years**							
High anxiety score[2]	Reference	/	/	/	10.1	Ref	Ref
	Looked After	/	/	/	18.7	2.0 (1.3-3.2)*	1.5 (1.0-2.5)
	Adopted	/	/	/	14.3	1.5 (1.0-2.2)*	1.4 (1.0-2.1)
High depression score[2]	Reference	/	/	/	9.5	Ref	Ref
	Looked After	/	/	/	18.5	2.2 (1.4-3.4)*	1.6 (1.0-2.5)*
	Adopted	/	/	/	12.2	1.3 (0.9-2.0)	1.3 (0.8-1.9)
Anxiety symptoms in past year	Reference	18.4	Ref	Ref	22.5	Ref	Ref
	Looked After	17.1	0.9 (0.4-1.9)	1.0 (0.5-2.2)	31.4	1.6 (1.1-2.3)*	1.4 (1.0-2.1)
	Adopted	17.8	1.0 (0.5-1.7)	1.0 (0.5-1.7)	25.8	1.2 (0.9-1.6)	1.2 (0.9-1.6)
Depression symptoms in past year	Reference	12.6	Ref	Ref	23.0	Ref	Ref
	Looked After	15.0	1.2 (0.6-2.7)	1.0 (0.4-2.2)	38.8	2.1 (1.5-3.0)*	1.6 (1.1-2.3)*
	Adopted	8.2	0.6 (0.3-1.4)	0.5 (0.2-1.2)	27.3	1.3 (0.9-1.7)	1.2 (0.9-1.6)

[1]Adjusted for age, relationship status, education, financial difficulties, social class, housing tenure.

For peer review only

[2]Adjusted for age, relationship status, education, financial difficulties, social class, housing tenure, parity, pregnancy intentional (pre-pregnancy models only), pregnancy status/intentions (5 year models only).
[3]Percentages rather than n given as these are results from imputed data, and so the n differs across the imputed datasets. The percentages shown are from results aggregated across all the imputed datasets using Rubin's rules.
*p<0.05

For peer review only

Table 5: Social support and criminal involvement outcomes by childhood public care status for men and women during pregnancy and up to when study child aged 5 years

		Men			Women		
		**%[3]**	**OR (95% CI)**		**%**	**OR (95% CI)**	
			Unadjusted	Adjusted[1]		Unadjusted	Adjusted[2]
**During pregnancy**							
Low social support	Reference	10.8	Ref	Ref	8.1	Ref	Ref
	Looked After	25.2	2.8 (1.4-5.4)*	2.2 (1.1-4.4)*	22.5	3.3 (2.1-5.0)*	1.6 (1.0-2.5)
	Adopted	8.7	0.8 (0.4-1.7)	0.7 (0.3-1.5)	10.5	1.3 (0.8-2.1)	1.2 (0.7-2.0)
Poor social network	Reference	12.7	Ref	Ref	8.8	Ref	Ref
	Looked After	26.4	2.5 (1.3-4.9)*	1.9 (1.0-3.8)	26.3	3.7 (2.5-5.5)*	2.0 (1.3-3.1)*
	Adopted	14.1	1.2 (0.6-2.1)	1.1 (0.6-2.1)	13.2	1.6 (1.0-2.3)*	1.5 (1.0-2.3)
**From pregnancy to child aged 5**							
Any criminal conviction	Reference	6.9	Ref	Ref	1.4	Ref	Ref
	Looked After	23.1	3.9 (1.5-10.4)*	2.6 (0.9-7.5)	6.2	4.4 (1.7-11.5)*	2.4 (0.9-6.5)
	Adopted	15.0	2.3 (1.1-5.0)*	1.8 (0.8-4.2)	4.6	3.2 (1.4-7.5)*	2.9 (1.2-7.1)*

[1]Adjusted for age, relationship status, education, financial difficulties, social class, housing tenure.
[2]Adjusted for age, relationship status, education, financial difficulties, social class, housing tenure, parity, pregnancy intentional (pre-pregnancy models only), pregnancy status/intentions (5 year models only).
[3]Percentages rather than n given as these are results from imputed data, and so the n differs across the imputed datasets. The percentages shown are from results aggregated across all the imputed datasets using Rubin's rules.
*p<0.05

For peer review only

Supplementary Tables

Table A: Social network questionnaire items in ALSPAC

Table B: Social support questionnaire items in ALSPAC

Table C: Summary of missing data

Table D: Summary of childhood and adolescent measures by care status for women and men

Tables E-I: Results for sensitivity analyses (complete case, pregnancy time-point outcomes only)

For peer review only

Social network and support measurement scales in ALSPAC

Below the items which comprise the social network scale and the social support scale are listed, along with the response options. Note that the ALSPAC data dictionary provides further details on these scales (see 'Mother Questionnaire D' document) and on all ALSPAC measures, and can be downloaded from the ALSPAC website: http://www.bristol.ac.uk/alspac/researchers/

Table A: Summary of ALSPAC social network questionnaire items

Questionnaire Items	Response Options
1. How many of your relatives and your partner's relatives do you see at least twice a year?	None, 1, 2-4, >4
2. About how many friends do you have?	None, 1, 2-4, >4
3. Overall, would you say you belong to a close circle of friends?	Yes, No
4. How many people are there that you can talk to about personal problems?	None, 1, 2-4, >4
5. How many people talk to you about their personal problems or their private feelings?	None, 1, 2-4, >4
6. If you have to make an important decision, how many people are there with whom you can discuss it?	None, 1, 2-4, >4
7. How many people are there among your family and friends from whom you could borrow £100 if you needed to?	None, 1, 2-4, >4
8. How many of your family and friends would help you in times of trouble?	None, 1, 2-4, >4
9. During the last month, how many times did you get together with one or more friends?	None, 1, 2-4, >4
10. During the last month, how many times did you get together with one or more of your relatives or your partner's relatives?	None, 1, 2-4, >4

Table B: Summary of ALSPAC social support questionnaire items

Questionnaire Items	Response Options
1. I have no one to share my feelings with	Exactly feel, often feel, sometimes feel, never feel
2. My partner provides the emotional support I need	Exactly feel, often feel, sometimes feel, never feel
3. There are other pregnant women with whom I can share my experiences	Exactly feel, often feel, sometimes feel, never feel
4. I believe in moments of difficulty my neighbours would help me	Exactly feel, often feel, sometimes feel, never feel
5. I'm worried that my partner might leave me	Exactly feel, often feel, sometimes feel, never feel
6. There is always someone with whom I can share my happiness and excitement about my pregnancy	Exactly feel, often feel, sometimes feel, never feel
7. If I feel tired I can rely on my partner to take over	Exactly feel, often feel, sometimes feel, never feel
8. If I was in financial difficulty I know my family would help if they could	Exactly feel, often feel, sometimes feel, never feel
9. If I was in financial difficulty I know my friends would help of they could	Exactly feel, often feel, sometimes feel, never feel
10. If all else fails I know the state will support and assist me	Exactly feel, often feel, sometimes feel, never feel

For peer review only

Table C: Summary of missing data

		N (%) missing	
		**Women (100%=8775)**	**Men (100%=3654)**
**Exposure variables**	Looked after status	588 (6.7)	0
	Adoption status	313 (3.6)	13 (0.4)
**Outcomes**			
(a) Pre-pregnancy (or pre-partner's pregnancy)	**Substance use**		
	Alcohol - frequency	182 (2.1)	99 (2.7)
	Smoking	145 (1.7)	142 (3.9)
	Cannabis	504 (5.7)	1771 (48.5)
	Ever had addiction	243 (2.8)	6 (0.2)
(b) Pregnancy (or partner's pregnancy)	**Mental health**		
	Depressive symptoms (EPDS)	799 (9.1)	134 (3.7)
	Anxiety symptoms (Crown-crisp)	884 (10.1)	134 (3.7)
	Ever had mental health problem	243 (2.8)	15 (0.4)
	**Social environment**		
	Social support	795 (9.1)	245 (6.7)
	Social network	422 (4.8)	188 (5.1)
(c) Child aged 5 years	**Substance use**		
	Alcohol - frequency	39 (0.4)	14 (0.4)
	Alcohol - binge	127 (1.5)	N/A
	Smoking	114 (1.3)	93 (2.6)
	Cannabis	52 (0.6)	47 (1.3)
	**Mental health**		
	Depressive symptoms (EPDS)	104 (1.2)	N/A
	Anxiety symptoms (Crown-crisp)	155 (1.8)	N/A
	Anxiety/nerves in past year	184 (2.1)	79 (2.2)
	Depression in past year	171 (1.9)	89 (2.4)
(d) Pregnancy (or partner's pregnancy) to child aged 5 years	Any criminal conviction	1681 (19.2)	1265 (34.6)
**Other**	Age	0	192 (5.3)
	Educational attainment	251 (2.9)	105 (2.9)
	Housing tenure	216 (2.5)	20 (0.6)
	Financial difficulties	481 (5.5)	115 (3.2)
	Social class	721 (8.2)	139 (3.8)
	Partner status	160 (1.8)	2 (0.1)
	Pregnancy with study child intentional	184 (2.1)	21 (0.6)
	Pregnancy intentions at 5 yrs	92 (1.1)	N/A
	Childhood happiness	93 (1.1)	44 (1.2)
	Pregnancy (own or partner's) pre 17yrs	272 (3.1)	88 (2.4)
	Suspended from school pre 17yrs	272 (3.1)	88 (2.4)
	In trouble with police pre 17yrs	272 (3.1)	88 (2.4)
	Number of schools attended	655 (7.5)	900 (24.6)

For peer review only

Table D: Summary of childhood and adolescent measures by care status for women and men

		Women			Men		
		Reference	Looked After	Adopted	Reference	Looked After	Adopted
Highest childhood happiness	Very happy (%)	75.4 (74.4-76.3)	46.9 (38.4-55.3)	72.9 (67.0-78.8)	77.9 (76.5-79.3)	61.9 (48.0-75.8)	77.2 (68.3-86.2)
Number of schools attended by 16 yrs	0-2 (%)	43.3 (42.2-44.4)	16.9 (10.0-23.8)	39.8 (33.0-46.6)	42.0 (40.2-43.8)	20.4 (6.2-34.6)	40.0 (28.1-52.0)
	5+ (%)	7.9 (7.3-8.4)	29.7 (21.6-37.9)	12.5 (8.1-16.9)	8.1 (7.1-9.1)	22.3 (8.2-36.5)	9.3 (2.2-16.4)
Suspended from school pre 17 years	Yes (%)	2.4 (2.1-2.7)	16.1 (9.7-22.4)	6.0 (2.6-9.3)	6.2 (5.4-7.0)	15.2 (4.5-25.9)	12.3 (5.2-19.4)
In trouble with police pre 17 years	Yes (%)	3.2 (2.8-3.6)	14.4 (8.3-20.5)	8.3 (4.4-12.1)	13.5 (12.3-14.6)	41.3 (27.0-55.6)	30.1 (20.3-40.0)
Pregnancy pre 17 years	Yes (%)	5.6 (5.1-6.1)	22.4 (15.2-29.6)	8.3 (4.5-12.2)	1.7 (1.3-2.2)	6.1 (0-13.1)	5.0 (0.2-9.8)

For peer review only

Table E: Characteristics of the women by care status in childhood, n=7088 (complete case, cross-sectional sample)

		Not looked after or adopted N=6794	Looked After (not adopted) N=110	Adopted N=184
Maternal age at delivery	Mean (years)	28.6	27.2	27.7
	<=23 years (%)	12.5 (11.7-13.3)	25.5 (18.1-34.6)	15.8 (11.1-21.8)
	>=34 years (%)	14.1 (13.3-14.9)	10.0 (5.6-17.3)	9.8 (6.2-15.1)
Relationship status	Husband (%)	82.1 (81.2-83.0)	62.7 (53.2-71.4)	78.8 (72.2-84.1)
	Resident partner (%)	13.8 (13.0-14.6)	21.8 (15.0-30.6)	15.2 (10.7-21.2)
	Non-resident partner (%)	3.6 (3.2-4.1)	13.6 (8.3-21.5)	4.9 (2.5-9.2)
	No partner (%)	0.4 (0.3-0.6)	1.8 (0.4-7.1)	1.1 (0.3-4.3)
Parity	0 (%)	44.6 (43.4-45.8)	29.1 (21.3-38.4)	48.9 (41.7-56.2)
	3+ (%)	4.6 (4.1-5.1)	21.0 (14.2-29.7)	7.1 (4.1-11.8)
This pregnancy intentional	Yes (%)	75.1 (74.1-76.2)	53.6 (44.2-62.9)	71.2 (64.2-77.3)
Highest maternal education	Degree (%)	14.0 (13.2-14.9)	3.6 (1.4-9.4)	8.2 (5.0-13.2)
	Vocational/none (%)	23.8 (22.8-24.9)	53.6 (44.2-62.9)	22.8 (17.3-29.5)
Financial difficulties	Q1 (none) (%)	39.0 (37.9-40.2)	17.3 (11.2-25.7)	26.6 (20.7-33.5)
	Q4 (high) (%)	17.4 (16.5-18.3)	36.4 (27.8-45.9)	19.6 (14.4-26.0)
Housing tenure	Owned/mortgaged (%)	81.5 (80.6-82.4)	43.6 (34.6-53.2)	75.5 (68.8-81.3)
Lowest social class of self and partner	I&II	26.5 (25.4-27.5)	17.3 (11.2-25.7)	25.5 (19.7-32.4)
	IV&V	18.9 (18.0-19.8)	26.4 (18.9-35.5)	23.9 (18.3-30.7)

For peer review only

Table F: Characteristics of the men by care status in childhood, n=2820 (complete case, cross-sectional sample)

		Not looked after or adopted N=2662	Looked After (not adopted) N=68	Adopted N=90
Paternal age at 18wks gestation	Mean (years)	30.9	29.6	29.2
	<=23 years (%)	7.6 (6.7-8.7)	16.2 (9.1-27.2)	12.2 (30.3-50.6)
	>=34 years (%)	29.0 (27.3-30.7)	20.6 (12.4-32.2)	20.0 (12.9-29.7)
Relationship status	Husband (%)	81.1 (79.6-82.5)	72.1 (60.0-81.6)	73.3 (63.1-81.6)
	Resident partner (%)	15.8 (14.4-17.2)	25.0 (16.0-36.9)	23.3 (15.6-33.4)
	Non-resident partner (%)	3.1 (2.5-2.9)	2.9 (0.7-11.4)	3.3 (1.1-10.0)
Highest education	Degree (%)	18.2 (16.8-19.7)	2.9 (0.7-11.4)	14.4 (8.5-23.5)
	Vocational/none (%)	28.1 (26.4-29.8)	54.4 (42.2-66.1)	31.1 (22.3-41.6)
Financial difficulties	Q1 (none) (%)	37.0 (35.2-38.9)	14.7 (8.0-25.5)	26.7 (18.4-36.9)
	Q4 (high) (%)	18.8 (17.3-20.3)	44.1 (32.6-56.3)	24.4 (16.5-34.6)
Housing tenure	Owned/mortgaged (%)	78.4 (76.8-80.0)	47.1 (35.3-59.2)	67.8 (57.3-76.8)
Lowest social class of self and partner	I&II	26.3 (24.6-28.0)	17.6 (10.2-28.9)	26.7 (18.4-36.9)
	IV&V	21.3 (19.7-22.9)	32.4 (22.1-44.6)	22.2 (14.7-32.2)

For peer review only

Table G: Substance use pre-pregnancy by childhood care status for men and women (complete case, cross-sectional sample)

		Men			Women		
		%	OR (95% CI)		%	OR (95% CI)	
**Outcome**	**Care status**		Unadjusted	Adjusted[1]		Unadjusted	Adjusted[2]
Smoked regularly	None	57.0	Ref	Ref	29.2	Ref	Ref
	Looked After	76.5	2.5 (1.4-4.3)*	1.4 (0.8-2.6)	61.8	3.9 (2.7-5.8)*	1.9 (1.3-3.0)*
	Adopted	64.4	1.4 (0.9-2.1)	1.2 (0.7-1.8)	41.8	1.7 (1.3-2.3)*	1.6 (1.1-2.2)*
Drank alcohol every day	None	26.7	Ref	Ref	11.1	Ref	Ref
	Looked After	17.6	0.6 (0.3-1.1)	0.8 (0.4-1.5)	7.3	0.6 (0.3-1.3)	0.8 (0.4-1.6)
	Adopted	20.0	0.7 (0.4-1.2)	0.7 (0.4-1.2)	21.2	2.2 (1.5-3.1)*	2.4 (1.7-3.5)*
Used cannabis	None	10.1	Ref	Ref	3.5	Ref	Ref
	Looked After	11.8	1.2 (0.6-2.5)	0.8 (0.3-1.7)	9.1	2.7 (1.4-5.3)*	1.6 (0.8-3.3)
	Adopted	17.8	1.9 (1.1-3.4)*	1.5 (0.8-2.8)	8.2	2.4 (1.4-4.2)*	2.0 (1.1-3.7)*
Has ever had addiction	None	4.3	Ref	Ref	0.8	Ref	Ref
	Looked After	14.7	3.8 (1.9-7.7)*	2.1 (1.0-4.5)	3.6	4.6 (1.6-13.0)*	2.3 (0.8-6.7)
	Adopted	7.8	1.9 (0.8-4.1)	1.6 (0.7-3.5)	2.2	2.7 (1.0-7.6)	2.3 (0.8-6.6)

[1]Adjusted for age, relationship status, education, financial difficulties, social class, housing tenure.

[2]Adjusted for age, relationship status, education, financial difficulties, social class, housing tenure, parity, pregnancy intentional.

*p<0.05

For peer review only

Table H: Mental health outcomes during the pregnancy by childhood care status for men and women (complete case, cross-sectional sample)

		Men			Women		
		%	**OR (95% CI)**		%	**OR (95% CI)**	
			Unadjusted	Adjusted[1]		Unadjusted	Adjusted[2]
High anxiety score (>6m, >9w)	None	10.6	Ref	Ref	10.4	Ref	Ref
	Looked After	14.7	1.5 (0.7-2.9)	1.3 (0.6-2.6)	26.4	3.1 (2.0-4.8)*	1.7 (1.1-2.7)*
	Adopted	11.1	1.1 (0.5-2.1)	1.0 (0.5-2.1)	19.0	2.0 (1.4-3.0)*	1.8 (1.2-2.6)*
High depression score (>8m, >13w)	None	12.7	Ref	Ref	8.6	Ref	Ref
	Looked After	22.1	2.0 (1.1-3.5)*	1.4 (0.8-2.6)	20.9	2.8 (1.8-4.5)*	1.5 (0.9-2.4)
	Adopted	12.2	1.0 (0.5-1.8)	0.9 (0.5-1.7)	16.3	2.1 (1.4-3.1)*	1.8 (1.2-2.8)*
Ever had mental health problem	None	8.3	Ref	Ref	10.0	Ref	Ref
	Looked After	17.6	2.4 (1.3-4.5)*	1.6 (0.8-3.2)	22.7	2.7 (1.7-4.2)*	1.7 (1.0-2.7)*
	Adopted	17.8	2.4 (1.4-4.2)*	2.2 (1.2-3.9)*	17.4	1.9 (1.3-2.8)*	1.8 (1.2-2.7)*

[1]Adjusted for age, relationship status, education, financial difficulties, social class, housing tenure.

[2]Adjusted for age, relationship status, education, financial difficulties, social class, housing tenure, parity, pregnancy intentional.

*p<0.05

For peer review only

Table I: Social support outcomes by childhood care status for men and women (complete case, cross-sectional sample)

		Men			Women		
	**Care status**	**%**	**OR (95% CI)**		**%**	**OR (95% CI)**	
			Unadjusted	Adjusted[1]		Unadjusted	Adjusted[2]
Low social support	None	13.9	Ref	Ref	8.0	Ref	Ref
	Looked After	26.5	2.2 (1.3-3.9)*	1.7 (1.0-3.0)	21.8	3.2 (2.0-5.1)*	1.5 (0.9-2.4)
	Adopted	15.6	1.1 (0.6-2.0)	1.1 (0.6-1.9)	9.8	1.2 (0.8-2.0)	1.1 (0.7-1.8)
Poor social network	None	15.8	Ref	Ref	8.6	Ref	Ref
	Looked After	22.1	1.5 (0.8-2.7)	1.0 (0.5-1.8)	24.5	3.5 (2.2-5.4)*	1.6 (1.0-2.6)*
	Adopted	15.6	1.0 (0.6-1.8)	0.9 (0.5-1.7)	13.6	1.7 (1.1-2.6)*	1.5 (1.0-2.4)

[1]Adjusted for age, relationship status, education, financial difficulties, social class, housing tenure.

[2]Adjusted for age, relationship status, education, financial difficulties, social class, housing tenure, parity, pregnancy intentional.

*p<0.05

For peer review only

STROBE 2007 (v4) Statement—Checklist of items that should be included in reports of *cohort studies*

Section/Topic	Item #	Recommendation	Reported on page #
**Title and abstract**	1	(*a*) Indicate the study's design with a commonly used term in the title or the abstract	1,2
		(*b*) Provide in the abstract an informative and balanced summary of what was done and what was found	2
**Introduction**			
Background/rationale	2	Explain the scientific background and rationale for the investigation being reported	4-5
Objectives	3	State specific objectives, including any prespecified hypotheses	2,4-5
**Methods**			
Study design	4	Present key elements of study design early in the paper	4-5
Setting	5	Describe the setting, locations, and relevant dates, including periods of recruitment, exposure, follow-up, and data collection	5
Participants	6	(*a*) Give the eligibility criteria, and the sources and methods of selection of participants. Describe methods of follow-up	5
		(*b*) For matched studies, give matching criteria and number of exposed and unexposed	N/A
Variables	7	Clearly define all outcomes, exposures, predictors, potential confounders, and effect modifiers. Give diagnostic criteria, if applicable	5-7
Data sources/ measurement	8*	For each variable of interest, give sources of data and details of methods of assessment (measurement). Describe comparability of assessment methods if there is more than one group	5-7
Bias	9	Describe any efforts to address potential sources of bias	7
Study size	10	Explain how the study size was arrived at	5
Quantitative variables	11	Explain how quantitative variables were handled in the analyses. If applicable, describe which groupings were chosen and why	5-7
Statistical methods	12	(*a*) Describe all statistical methods, including those used to control for confounding	7
		(*b*) Describe any methods used to examine subgroups and interactions	N/A
		(*c*) Explain how missing data were addressed	7
		(*d*) If applicable, explain how loss to follow-up was addressed	7
		(*e*) Describe any sensitivity analyses	7
**Results**			

For peer review only

Participants	13*	(a) Report numbers of individuals at each stage of study—eg numbers potentially eligible, examined for eligibility, confirmed eligible, included in the study, completing follow-up, and analysed	5,7
		(b) Give reasons for non-participation at each stage	5
		(c) Consider use of a flow diagram	N/A
Descriptive data	14*	(a) Give characteristics of study participants (eg demographic, clinical, social) and information on exposures and potential confounders	8, Tables 1, 2 and B
		(b) Indicate number of participants with missing data for each variable of interest	Table A
		(c) Summarise follow-up time (eg, average and total amount)	5
Outcome data	15*	Report numbers of outcome events or summary measures over time	8-9
Main results	16	(*a*) Give unadjusted estimates and, if applicable, confounder-adjusted estimates and their precision (eg, 95% confidence interval). Make clear which confounders were adjusted for and why they were included	Tables 3-5
		(*b*) Report category boundaries when continuous variables were categorized	5-7
		(*c*) If relevant, consider translating estimates of relative risk into absolute risk for a meaningful time period	N/A
Other analyses	17	Report other analyses done—eg analyses of subgroups and interactions, and sensitivity analyses	9
**Discussion**			
Key results	18	Summarise key results with reference to study objectives	9-10
**Limitations**			
Interpretation	20	Give a cautious overall interpretation of results considering objectives, limitations, multiplicity of analyses, results from similar studies, and other relevant evidence	10-12
Generalisability	21	Discuss the generalisability (external validity) of the study results	12
**Other information**			
Funding	22	Give the source of funding and the role of the funders for the present study and, if applicable, for the original study on which the present article is based	17

*Give information separately for cases and controls in case-control studies and, if applicable, for exposed and unexposed groups in cohort and cross-sectional studies.

Note: An Explanation and Elaboration article discusses each checklist item and gives methodological background and published examples of transparent reporting. The STROBE checklist is best used in conjunction with this article (freely available on the Web sites of PLoS Medicine at http://www.plosmedicine.org/, Annals of Internal Medicine at http://www.annals.org/, and Epidemiology at http://www.epidem.com/). Information on the STROBE Initiative is available at www.strobe-statement.org.

For peer review only
